# An RNA modification enzyme directly senses reactive oxygen species for translational regulation in *Enterococcus faecalis*

**DOI:** 10.1038/s41467-023-39790-x

**Published:** 2023-07-11

**Authors:** Wei Lin Lee, Ameya Sinha, Ling Ning Lam, Hooi Linn Loo, Jiaqi Liang, Peiying Ho, Liang Cui, Cheryl Siew Choo Chan, Thomas Begley, Kimberly Ann Kline, Peter Dedon

**Affiliations:** 1 Antimicrobial Resistance IRG, Singapore MIT Alliance for Research and Technology, Singapore, Singapore; 2 School of Biological Sciences, Nanyang Technological University, Singapore, Singapore; 3 Singapore Centre for Environmental Life Sciences Engineering, Nanyang Technological University, Singapore, Singapore; 4 Department of Biological Sciences and The RNA Institute, University at Albany, Albany, NY USA; 5 Department of Microbiology and Molecular Medicine, Faculty of Medicine, University of Geneva, Geneva, Switzerland; 6 Dept. of Biological Engineering, Massachusetts Institute of Technology, Cambridge, MA USA; 7 Present Address: Helmholtz-Zentrum für Infektionsforschung GmbH, Inhoffenstraße 7, 38124 Braunschweig, Germany; 8 Present Address: Department of Oral Biology, University of Florida College of Dentistry, Gainesville, FL USA; 9 Present Address: School of Chemistry, Chemical Engineering and Biotechnology, College of Engineering, Nanyang Technological University, Singapore, Singapore; 10 Present Address: Critical Analytics for Manufacturing Personalized-Medicine IRG, Singapore MIT Alliance for Research and Technology, Singapore, Singapore

**Keywords:** Bacterial genetics, Non-coding RNAs

## Abstract

Bacteria possess elaborate systems to manage reactive oxygen and nitrogen species (ROS) arising from exposure to the mammalian immune system and environmental stresses. Here we report the discovery of an ROS-sensing RNA-modifying enzyme that regulates translation of stress-response proteins in the gut commensal and opportunistic pathogen *Enterococcus faecalis*. We analyze the tRNA epitranscriptome of *E. faecalis* in response to reactive oxygen species (ROS) or sublethal doses of ROS-inducing antibiotics and identify large decreases in N^2^-methyladenosine (m^2^A) in both 23 S ribosomal RNA and transfer RNA. This we determine to be due to ROS-mediated inactivation of the Fe-S cluster-containing methyltransferase, RlmN. Genetic knockout of RlmN gives rise to a proteome that mimics the oxidative stress response, with an increase in levels of superoxide dismutase and decrease in virulence proteins. While tRNA modifications were established to be dynamic for fine-tuning translation, here we report the discovery of a dynamically regulated, environmentally responsive rRNA modification. These studies lead to a model in which RlmN serves as a redox-sensitive molecular switch, directly relaying oxidative stress to modulating translation through the rRNA and the tRNA epitranscriptome, adding a different paradigm in which RNA modifications can directly regulate the proteome.

## Abstract (web summary)

Here the authors found an RNA modifying enzyme that serves as a molecular switch, directly relaying the sensing of reactive oxygen species to chemical modifications in rRNA and tRNA for increasing stress response proteins in the proteome.

## Introduction

Reactive oxygen species (ROS) such as superoxide (O_2_^−^) and hydrogen peroxide (H_2_O_2_) play fundamental roles in shaping bacterial evolution^1^. Bacteria are exposed to ROS from a variety of sources, endogenously as byproducts of aerobic respiration and exogenously from redox-active natural products and the respiratory/oxidative bursts of activated mammalian immune cells^2^. If not neutralized, ROS damage essential cellular components including DNA, lipids, carbohydrates, and proteins^1^. Bacteria have thus evolved ROS defense systems such as superoxide dismutases (SOD), catalases, glutathione, thioredoxin systems, peroxidases, and nitrate/nitrite reductases^3^. These defenses are often regulated transcriptionally by ROS-sensing transcription factors such as OxyR, PerR, OhrR, and SoxR^4–6^.

Recent evidence points to mechanisms of translational regulation of bacterial stress response systems. For example, the hypoxic stress response in mycobacteria involves reprogramming of dozens of modified ribonucleosides on tRNAs—the tRNA epitranscriptome—to cause selective translation of codon-biased mRNAs from hypoxia response genes including the DosR transcription factor and its regulon^7^. Modifications on other forms of RNA also participate in diverse cellular processes by altering RNA stability, structure, localization, and protein-RNA interactions^8^.

Here we report the discovery of an ROS-sensing RNA-modifying enzyme that regulates translation of stress-response proteins in the gut commensal and opportunistic pathogen *Enterococcus faecalis*. Following exposure to the superoxide generator, menadione, or sublethal doses of ROS-inducing erythromycin and chloramphenicol, analysis of 24 modified ribonucleosides of the epitranscriptome revealed large decreases in N^2^-methyladenosine (m^2^A) in both 23 S ribosomal RNA and transfer RNA possibly caused by ROS-mediated inactivation of the Fe-S cluster-containing methyltransferase, RlmN. Loss of RlmN altered protein expression in a way that mimicked menadione exposure, such as increased superoxide dismutase and decreased virulence proteins. These studies suggest that RlmN acts as a redox-sensitive molecular switch that links environmental and antibiotic-induced ROS exposure to epitranscriptome dynamics in ribosomal and transfer RNA to effect translation of stress response proteins.

## Results

While transcriptional regulation in response to stress is well established in bacteria, translational regulation is less well understood. Our demonstration of hypoxia-induced epitranscriptome reprogramming and codon-biased translation in mycobacteria^7^ led us to hypothesize that a similar mechanism might hold true for the response of *Enterococcus faecalis* to the stress of antibiotic exposure. Here we quantified 24 modifications in the rRNA and tRNA in two *E. faecalis* strains: OG1RF, a strain derived from the human commensal oral isolate OG1^9^, and V583^10^, a multidrug resistant clinical isolate. V583 possesses an erythromycin resistance methyltransferase (ErmB) that methylates the N^6^-position of adenosine (m^6^A, m^6,6^A) in 23 S rRNA at position 2058 (*Escherichia coli* numbering)^11^ and prevents binding of macrolides (e.g., erythromycin), lincosamides, and streptogramin B (MLS)^11^, but only confers partial resistance to erythromycin^12^. OG1RF lacks ErmB and is thus about 100-fold more sensitive to erythromycin than V583.

**Fig. 1:**
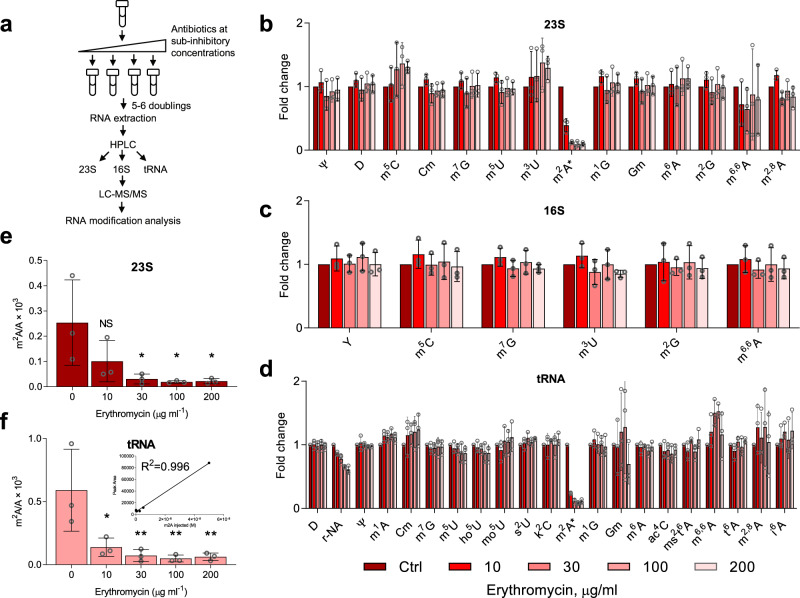
Epitranscriptomic profiling of 23 S and 16 S rRNA and tRNA of *Enterococcus faecalis* V583 grown in the presence of growth-permissive concentrations of erythromycin. **a** Epitranscriptome profiling workflow. Log-phase cultures of V583 were diluted with growth medium containing erythromycin below its MIC (10–200 µg/mL) and allowed to grow for 5-6 doublings to mid-log phase, after which RNA was isolated and RNA modifications quantified by LC-MS/MS. **b**–**d** Changes in the levels of RNA modifications in V583 at varying doses of erythromycin (key at bottom of **d**) compared to untreated cells. Modification levels are shown as fold-change relative to untreated control for 23 S rRNA (**b**), 16 S rRNA (**c**), and tRNA (**d**). Modifications are arranged from left to right in ascending retention time. See Supplementary Table 1 for names, retention times, and precursor and product-ion masses for the RNA modifications. Error bars represent mean ± SD for fold-change values calculated from 3 independent measurements of RNA modification normalized signal intensity in the mutant strain divided by the average signal intensity for three wild-type samples. **e**, **f** Effect of erythromycin dose on the ratio of m^2^A to adenosine. Ribonucleosides were quantified using LC-MS calibration curves, as illustrated for tRNA in the inset. All data are derived from 3 experiments (mean ± SD, *n* = 3). Statistical analysis by one-way analysis of variance (ANOVA) with Dunnett’s test versus untreated controls; *P < 0.05; **P < 0.005. **e** Exact *p* values: 30 μg/ml, 0.0291; 100 μg/ml, 0.0220; 200 μg/ml 0.0235. **f** Exact *p* values: 10 μg/ml, 0.0146; 30 μg/ml, 0.0062; 100 μg/ml, 0.0047; 200 μg/ml, 0.0055.

We first explored antibiotic effects on V583’s epitranscriptome by growing cells in the presence of sub-inhibitory concentrations of erythromycin (10–200 μg/mL; Fig. 1a). Following purification of 23 S and 16 S rRNAs and tRNA and hydrolysis to ribonucleosides, 24 modified ribonucleosides were quantified by chromatography-coupled mass spectrometry (LC-MS) in each type of RNA (Fig. 1b–d, Supplementary Table 1)^7^. Neither m^6^A nor m^6,6^A increased in 23 S rRNA with erythromycin treatment, confirming that Erm expression in V583 is constitutive^12^. While most of the monitored modifications remained relatively unchanged with treatment, a striking reduction in 2-methyladenosine (m^2^A) in both 23 S rRNA and tRNA was observed for erythromycin exposure (Fig. 1b–d), with dose-dependency (Fig. 1e, f).

**Fig. 2:**
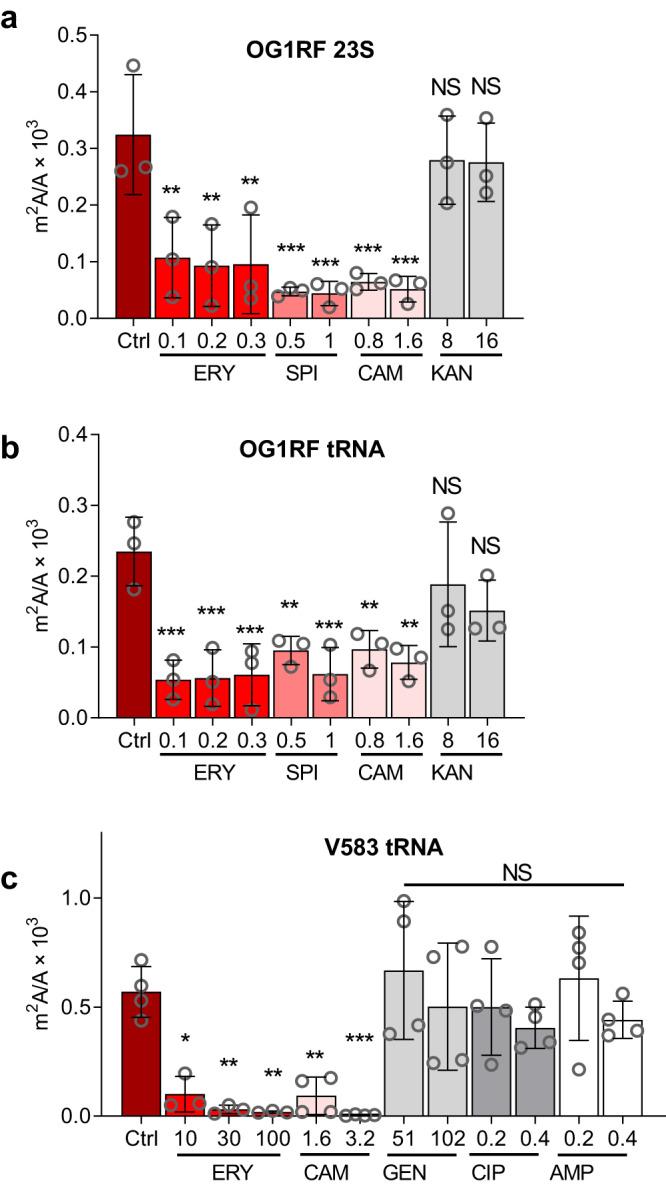
Reduction of m2A in V583 and OG1RF is specific to bacteriostatic macrolides and chloramphenicol. OG1RF (**a**, **b**) and V583 (**c**) were exposed to antibiotics at concentrations below their minimal inhibitory concentrations (MICs), and the quantity of m^2^A in tRNA and 23 S rRNA measured by LC-MS. m^2^A levels decreased for the bacteriostatic macrolides erythromycin (ERY; **a**–**c**) and spiramycin (SPI; **b**) as well as chloramphenicol (CAM; **a**–**c**), but not for bactericidal ciprofloxacin (CIP; **c**), ampicillin (AMP; **c**), or the aminoglycosides gentamicin (GEN; **c**) and kanamycin (KAN; **a**, **b**). Data represent mean ± SD, for *n* = 4 (**c**) and *n* = 3 (**a**, **b**) independent experiments. Statistical analysis by one-way analysis of variance (ANOVA) with Dunnett’s test versus untreated controls. NS not significant; *P < 0.05; **P < 0.005; ***P < 0.0005. **a** Exact *p* values: 0.1 ERY, 0.0036; 0.2 ERY, 0.0020; 0.3 ERY, 0.0022; 0.5 SPI, 0.0003; 1 SPI, 0.0003; 0.8 CAM, 0.0006; 1.6 CAM, 0.0003. **b** Exact *p* values: 0.1 ERY, 0.0005; 0.2 ERY; 0.0006; 0.3 ERY, 0.0007; 0.5 SPI, 0.0064; 1.0 SPI, 0.0008; 0.8 CAM, 0.0071; 1.6 CAM, 0.0022. **c** Exact *p* values: 10 ERY, 0.0149; 30 ERY, 0.0039; 100 ERY, 0.0031; 1.6 CAM, 0.0063; 3.2 CAM, 0.0009.

To assess if the m^2^A reduction was unique to V583, we repeated the study with *E. faecalis* OG1RF at growth-permissive erythromycin concentrations (0.1–0.3 μg/mL; Supplementary Table 2). We again observed a significant concentration-dependent decrease in m^2^A in both 23 S rRNA and tRNA (Fig. 2a, b), with insignificant changes in the other 23 modifications (Supplementary Fig. 3). We next asked if m^2^A reduction was unique to erythromycin or a general response to all antibiotics. At 10–25% of the MICs of the different antibiotics (Supplementary Table 2), m^2^A was reduced by macrolides erythromycin and spiramycin and the phenicol antibiotic chloramphenicol, all of which are bacteriostatic, but not by the bactericidal ciprofloxacin, ampicillin, or aminoglycosides kanamycin and gentamicin (Fig. 2a–c). Macrolides and chloramphenicol bind to the large (50 S) ribosomal subunit at the peptide exit tunnel and peptidyl transferase center, respectively, while aminoglycosides target the small (30 S) ribosomal subunit, in contrast to ciprofloxacin and ampicillin as gyrase inhibitor and cell wall disruptor, respectively^13^. The antibiotic-induced reduction of m^2^A is less in OG1RF compared to V583 (Fig. 2c), most likely due to the markedly lower concentrations of erythromycin and chloramphenicol used with OG1RF. So far, the data reveal that exposure of *E. faecalis* to sub-inhibitory concentrations of antibiotics that target the 50 S ribosomal subunit selectively reduce m^2^A in 23 S rRNA and tRNA, which raises the question of the mechanistic basis for this epitranscriptome behavior.

**Fig. 3:**
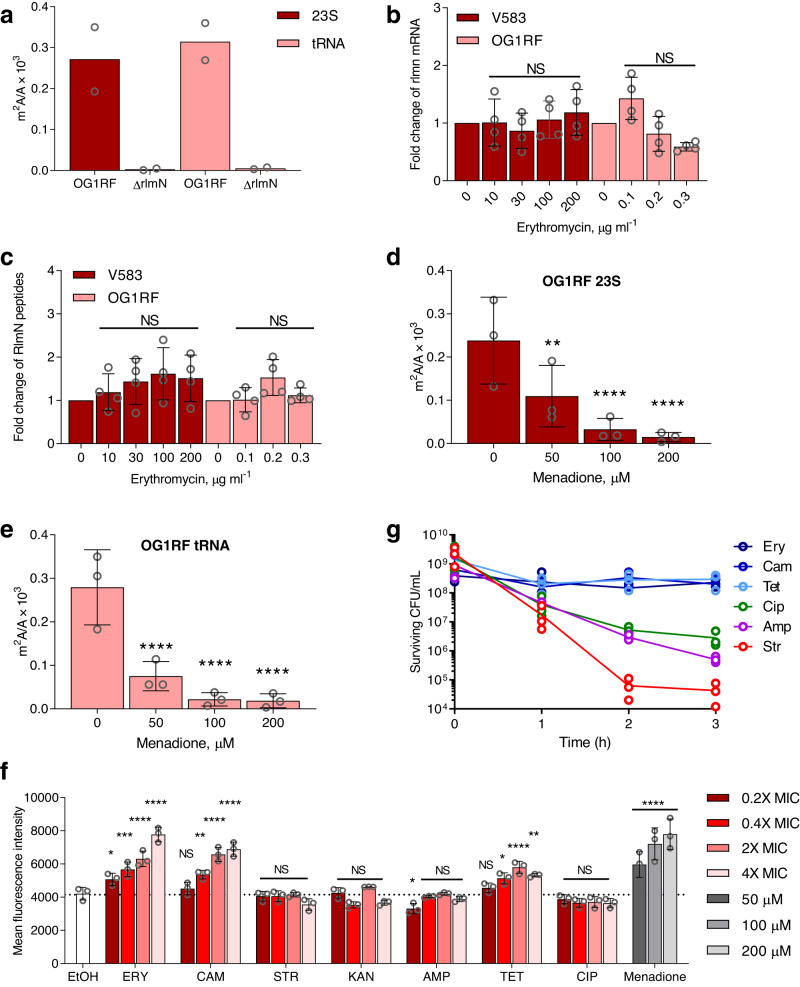
RlmN is regulated at the protein level by reactive oxygen species ROS. **a** Loss of *rlmN* abolishes m^2^A in OG1RF (mean ± SD, *n* = 2). **b** RT-qPCR of *rlmN* in OG1RF and V583 upon erythromycin treatment. Data represent mean ± SD for 4 experiments: two biological replicates analyzed with two different primer sets. Statistical analysis by one-way analysis of variance (ANOVA) with Dunnett’s test vs. untreated: NS not significant. **c** Targeted proteomics of RlmN in OG1RF and V583 upon erythromycin treatment. Data represent mean ± SD for six experiments: three peptides monitored in two independent experiments. Statistical analysis by one-way analysis of variance (ANOVA) with Dunnett’s test vs. untreated: NS not significant. Ratio of m^2^A to A in **d** 23 S rRNA and **e** tRNA with menadione treatment. Data represent mean ± SD for three independent experiments. Statistical analysis by one-way analysis of variance (ANOVA) with Dunnett’s test versus untreated: **P < 0.005; ****, *P* < 0.0001. **d** Exact *p* values: 50 μM, 0.0886; 100 μM, 0.0102; 200 μM, 0.0064. **e** Exact *p* values: 50 μM, 0.0020; 100 μM, 0.0004; 200 μM, 0.0004. **f** Mean fluorescence intensity of CellROX Green Dye+ in OG1RF treated with antibiotics at indicated concentrations; MIC minimal inhibitory concentration. Data represent mean ± SD for three independent experiments. Statistical analysis by two-way analysis of variance (ANOVA) with Dunnett’s test versus EtOH; NS not significant; *P* < 0.05, *P* < 0.005, *P* < 0.0005, and *P* < 0.0001 are denoted as *, **, ***, and **** respectively. Exact *p* values: ERY 0.2× MIC, 0.0432; ERY 0.4× MIC, 0.0002; ERY 2× MIC, <0.0001; ERY 4× MIC, <0.0001; CAM 0.4× MIC, 0.0043; CAM 2× MIC, <0.0001; CAM 4× MIC, <0.0001; AMP 0.2× MIC, 0.0343; TET 0.4× MIC, 0.0285; TET 2× MIC, <0.0001; TET 4× MIC, 0.0063; Menadione 50 μM, <0.0001; Menadione 100 μM, <0.0001; Menadione 200 μM, <0.0001. **g** Cell killing kinetics of various antibiotics at 10× MIC reveal that ROS-generating antibiotics are not bactericidal in OG1RF. Symbols represent individual data points for three independent experiments. Source data are provided as a Source Data file. EtOH ethanol, ERY erythromycin, CAM chloramphenicol, STR streptomycin, KAN kanamycin, AMP ampicillin, TET tetracycline, CIP ciprofloxacin.

In *Escherichia coli*, m^2^A formation is catalyzed by RNA methyltransferase RlmN, which methylates A2503 in 23 S rRNA at the peptidyl transferase center in the 50 S ribosomal subunit and A37 in the subset of tRNAs bearing adenine at this position in the anticodon stem loop^11^. Since RlmN in *E. faecalis* has not been previously characterized, we analyzed m^2^A levels in a *ΔrlmN* deletion mutant in OG1RF and found complete loss of the modification in 23 S rRNA and tRNA (Fig. 3a). We first asked if the reduction of m^2^A by macrolides and chloramphenicol involved transcriptional or translational regulation of RlmN. Neither the level of *rlmN* mRNA (qPCR) nor the level of RlmN protein (targeted proteomics) was affected significantly by erythromycin treatment (Fig. 3b, c). These data suggested that the activity of RlmN was regulated post-translationally by antibiotic exposure.

Among possible mechanisms for regulating RlmN activity, one involves antibiotic-induced oxidative stress. RlmN is a radical S-adenosylmethionine (SAM) enzyme containing a [4Fe-4S] cluster that is sensitive to disruption by reactive oxygen species (ROS)^2^. To test this model, we grew OG1RF in the presence of the superoxide radical generator, menadione^14^, at sub-inhibitory concentrations and then measured m^2^A in 23 S rRNA and tRNA. Menadione treatment caused a dose-dependent decrease in m^2^A in both rRNA and tRNA (Fig. 3d, e). This result implies that the macrolides and chloramphenicol also induced ROS in OG1RF and V583, which we assessed using the fluorogenic superoxide-specific probe CellROX Green to quantify antibiotic-induced ROS levels in OG1RF^15^. As expected, both erythromycin and chloramphenicol increased CellROX Green fluorescence at sub-inhibitory and higher concentrations (Fig. 3f). Given the impact of antibiotics on bacteria cell shape and size that may lead to artifacts on the interpretation of flow cytometric analyses of ROS-detecting fluorescent dyes and the importance of setting FSC and SSC gates^16^, we confirmed that the fluorescence changes were due to ROS using an optimized gating and other flow cytometric parameters that minimize artifacts in dye-based ROS detection (Supplementary Figs. 5, 6)^15^. Indeed, two observations confirm the absence of size artifacts. First, erythromycin reduced mean FSC-H and SSC-H values at sub-inhibitory concentrations (Supplementary Fig. 6), suggesting smaller cell size, yet mean fluorescence intensity of CellROX Green Dye increased directly with erythromycin concentration (Fig. 3f ). On the other hand, ampicillin caused increases in the mean FSC-H and SSC-H (Supplementary Fig. 6) but did not cause an increase in mean fluorescence intensity of CellROX Green Dye (Fig. 3f ). These two observations convincingly show that antibiotic-induced changes in fluorescence in the CellROX dye studies were due to ROS activation of the dye and not flow cytometry size artifacts. Not all ribosome-targeting antibiotics generate ROS in OG1RF. Sub- and supra-inhibitory concentrations of tetracycline, which binds to the 30 S subunit, induced an increase in CellROX Green fluorescence whereas the aminoglycosides streptomycin and kanamycin did not (Fig. 3f). Further, the mechanistically distinct ampicillin and ciprofloxacin also did not increase CellROX Green fluorescence at both sub- or supra-inhibitory concentrations (Fig. 3f). These observations would seem to contradict Léger et al., who reported that the β-lactams amoxicillin and cefotaxime caused superoxide production by reduction of demethylmenaquinone (DMK) in *E. faecalis*^17^. However, they did not measure intracellular superoxide. It is well established that *E. faecalis* produces high levels of extracellular superoxide by way of externally-facing, membrane-bound DMK, but the superoxide is unable to diffuse through cell walls and be detected as intracellular superoxide by CellRox and is also rapidly dismutated to hydrogen peroxide outside the cell^18^; Léger et al. measured extracellular hydrogen peroxide as their surrogate for superoxide^17^. That RlmN activity observed here could be caused by antibiotic-induced reduction in SAM levels is not possible given the lack of change in methylation-based modifications other than m^2^A. Further, while we did not measure tRNA levels here, the constancy of all other RNA modification levels confirms that antibiotic treatment did not significantly change the levels of the rRNA and tRNA substrates of RlmN. Though we our studies do not provide proof of a direct reaction of ROS with RlmN, the dose-response for RlmN inhibition with menadione- (Fig. 3e), erythromycin- (Figs. 2c, 3f), and chloramphenicol-induced ROS (Figs. 2c, 3f) strongly suggests that RlmN is inactivated by intracellular superoxide resulting from exposure of *E. faecalis* to macrolides and chloramphenicol.

Given the controversial model that bactericidal antibiotics share a common mechanism of generating cytotoxic ROS^15,19,20^, we tested antibiotics for their bactericidal and bacteriostatic activity in *E. faecalis*. Erythromycin and chloramphenicol have been classified as bacteriostatic^21^, with bactericidal activity at high concentrations against *Streptococcus pneumoniae*^21^, while ciprofloxacin, ampicillin, and streptomycin are classified as bactericidal^21^. However, our data show that erythromycin and chloramphenicol induce superoxide production at low concentrations in OG1RF, while ciprofloxacin, ampicillin, and streptomycin do not. Léger et al. observed ROS generation by the related β-lactam, amoxicillin, at supra-lethal concentrations, again by measuring hydrogen peroxide which was likely generated from superoxide produced extracellularly. To establish bactericidal activity, we performed killing assays with OG1RF in the presence of antibiotics at ten times their MIC and found that antibiotics that increase CellROX Green fluorescence (*i.e*., erythromycin, chloramphenicol, tetracycline) are bacteriostatic in OG1RF, while ciprofloxacin, ampicillin, and streptomycin, which do not induce ROS, are bactericidal (Fig. 3g). These data not only further disprove the link between bactericidal antibiotics, ROS, and cell death, but also raise the question of the role of RlmN in *E. faecalis* antibiotic sensitivity.

**Fig. 4:**
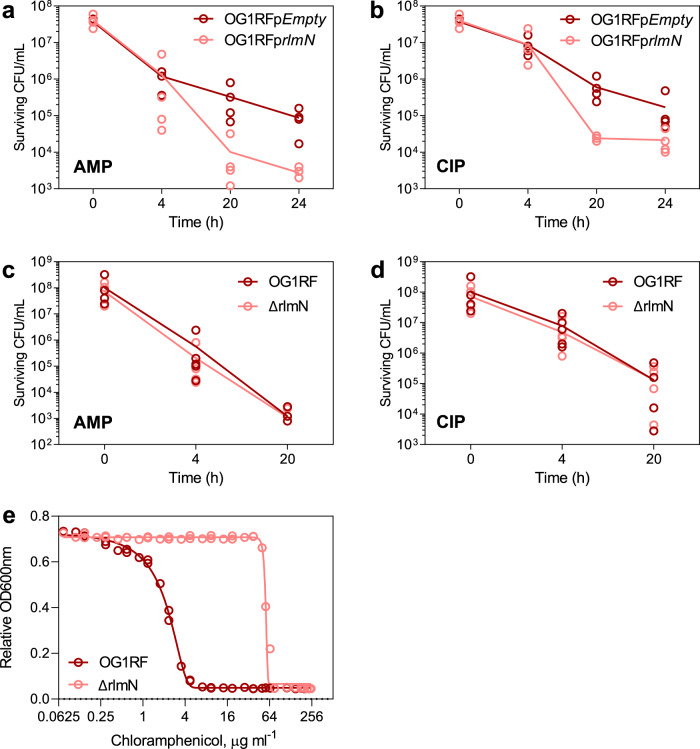
Phenotypic characterization of rlmN KO (ΔrlmN) and over-expressed RlmN (OG1RFprlmN). Kinetics of cell killing for OG1RFp*Empty* and OG1RFp*rlmN* grown with 5× MIC for **a** ampicillin (5 µg/mL) and **b** 5 µg/mL ciprofloxacin. Kinetics of cell killing for OG1RF and Δ*rlmN* grown with 5× MIC for **c** 5 µg/mL ampicillin and **d** 5 µg/mL ciprofloxacin. Graphs show individual data for 4 independent experiments. **e** Growth assay for minimum inhibitory concentration (MIC) of chloramphenicol with OG1RF and Δ*rlmN*. The graph shows data from 4 biological replicates.

We next investigated the effect of RlmN activity on *E. faecalis* antibiotic sensitivity, using the *ΔrlmN* deletion mutant and a strain over-expressing RlmN. Here we created a constitutive over-expression mutant, OG1RFp*rlmN*, which carried an RlmN-expressing plasmid under the constitutive Sortase A promoter in vector pGCP123^22^; and a control strain, OG1RFp*Empty*, which carried the same plasmid lacking the coding sequence for RlmN. Over-expression of RlmN did not affect the MIC for erythromycin, streptomycin, kanamycin, ampicillin, tetracycline, or ciprofloxacin (Supplementary Table 2), but did increase the sensitivity of OG1RFp*rlmN* to killing by the bactericidal antibiotics ampicillin and ciprofloxacin by 10-fold (Fig. 4a, b). Loss of RlmN, however, had no effect on *E. faecalis* growth in the presence of up to 5-times the wild-type MIC for the 5 antibiotics (Supplementary Table 2, Fig. 4c, d), except for a 16-fold increase in MIC for chloramphenicol (Fig. 4e, Supplementary Table 3). Taken together, (1) over-expression of RlmN increases *E. faecalis* sensitivity to ampicillin and ciprofloxacin and (2) loss of RlmN activity confers resistance to chloramphenicol suggesting that RlmN could play a role in regulating antibiotic tolerance.

**Fig. 5:**
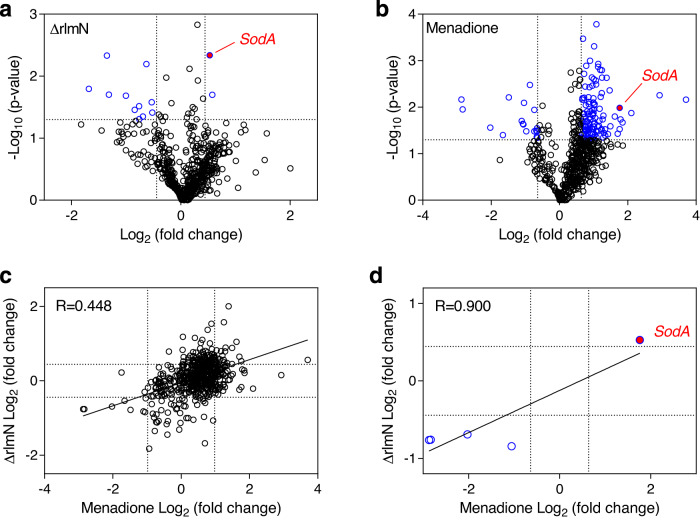
Loss of rlmN and treatment with menadione cause similar changes in the OG1RF proteome. Volcano plots showing changes in protein levels in OG1RF caused by **a** *rlmN* knockout (Δ*rlmN*) and **b** menadione treatment. *P* values were calculated using the two-sided Student’s *t* test. The log_2_ fold-change ( *x* axis) was plotted against the −log_10_( *p* value) ( *y* axis). A −log_10_( *p* value) of 1.30 threshold (*P* < 0.05) is denoted by the dotted horizontal line, while the vertical dotted lines represent fold-change values at ±1 SD. **c** Proteins detected in both menadione-treated and Δ*rlmN* without a *P* value cutoff. Pearson correlation *R* = 0.448, two-tailed *p* value < 0.0001. **d** Proteins that changed significantly (*P* < 0.05) in both menadione-treated and *ΔrlmN* cells. Cutoff was set at 1 SD, which is log_2_(fold-change) of ±0.639 for menadione treatment and log_2_(fold-change) of ±0.443 for Δ*rlmN*. Blue circles represent proteins that significantly up- or downregulated in both, relative to untreated, wild-type cells. These proteins are shown in Supplementary Table 4. Data shown are for three biological replicates. Red circles denote superoxide dismutase (SodA). Pearson correlation *R* = 0.900, two-tailed *p* value 0.0372.

We next defined the effects of RlmN activity on the cell proteome. Our results establish that RlmN’s methyltransferase activity is attenuated by superoxide, raising the possibility that RlmN serves as a redox sensor that regulates cell stress response. To test this idea, we performed quantitative proteomics using multiplexed isobaric Tandem Mass Tags (TMT) to identify differentially expressed proteins between OG1RF, Δ*rlmN*, and OG1RF grown in menadione (Fig. 5a, b; Supplementary Table 3; Supplementary Data 1). Only a few proteins were upregulated in Δ*rlmN* as compared to OG1RF treated with menadione, suggesting precision of RlmN as a molecular switch. Indeed, we found a positive correlation of *R* = 0.900 between statistically significant (*p* < 0.05) protein changes in Δ*rlmN* and OG1RF treated with menadione (Fig. 5d). One of these was the antioxidant defense enzyme, superoxide dismutase, for which there is a single gene (*sodA*) in *E. faecalis*^23^. Of note, another well-known antioxidant defense enzyme, catalase (KatA), was not consistently detected in the proteomic analyses (Supplementary Data 1). Interestingly, the common set of proteins that are downregulated in both datasets include proteins associated with virulence (Supplementary Table 4). Pilus subunit protein EbpA is a major virulence factor in *E. faecalis*, involved in biofilm formation, endocarditis, and catheter-associated urinary tract infection^24^; and WxL domain-containing proteins^25,26^, which, in *Enterococus faecium*, have been implicated in survival in bile salt and the pathogenesis of endocarditis^27^.

## Discussion

**Fig. 6:**
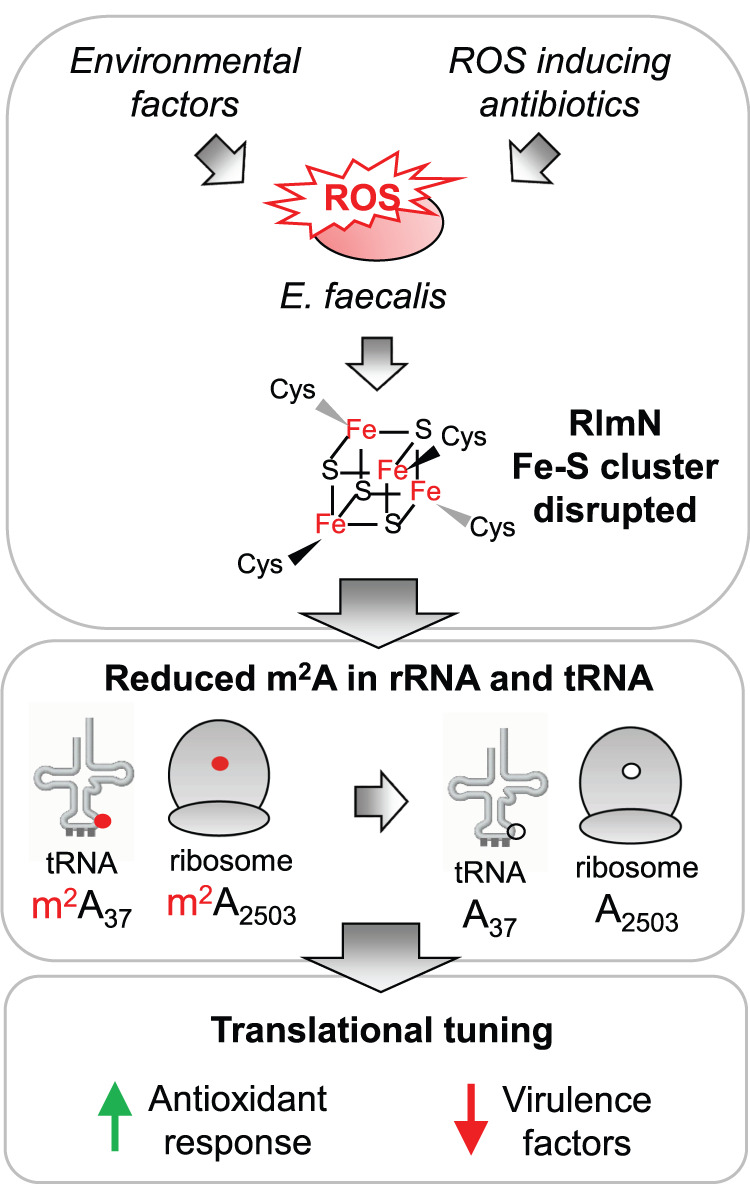
Working model of RlmN as a sensor for oxidative stress. Environmental factors as well as certain antibiotics induce reactive oxygen species in *Enterococcus faecalis*. These lead to the inactivation of RlmN through perturbation of its Fe-S cluster, exerting dual effects on both the ribosome and tRNA. m^2^A (shown in red) is present in position 37 of selected tRNAs and 23 S rRNA in the peptidyl transferase center of the ribosome. Loss of m^2^A modifications on the rRNA and tRNA leads to a modified pool of ribosomes and tRNA, which lead to the upregulation of antioxidant response and downregulation of virulence factors for improved survival in the presence of antibiotics.

Based on the results presented here, we propose a mechanism (Fig. 6) in which RlmN serves as a ROS-sensitive molecular switch that modulates physiological responses to oxidative stress and confers phenotypic resistance to environmental stresses and antimicrobial agents. This is perhaps not surprising given the importance of other Fe-S proteins, such as SoxR, Fnr, and aconitase, as ROS sensors linked to changes in gene expression and cell phenotype^6^. RlmN and m^2^A are absent in eukaryotes and, within prokaryotes, RlmN is the only enzyme that synthesizes m^2^A. The inertness of the C2 of adenosine to electrophilic attack and the low acidity of the C2 proton requires a free radical SAM intermediate. This mechanism, distinctly different from typical SAM-dependent methyltransferases, is unique to RlmN^28^ and the chloramphenicol-florfenicol resistance methyltransferase (Cfr)^29^. Here we showed that RlmN activity is not only strongly dependent upon intracellular superoxide levels but also regulates levels of SodA, which promotes antibiotic tolerance^30^ and facilitates survival in macrophages^23^ in *E. faecalis* and other bacteria.

So how does exposure of *E. faecalis* to ribosome-binding antibiotics lead to elevated superoxide levels? While there is no universal mechanism by which antibiotic exposure causes increases in ROS in bacteria, in spite of earlier claims^20^, there are numerous pathways for generating superoxide and other ROS and for environmental exposure of bacteria to ROS^2^. *E. faecalis* generates large amounts of extracellular superoxide^18^, with Léger et al. showing that supra-lethal ampicillin doses increase these levels^17^. While we did not measure extracellular ROS, superoxide cannot diffuse through the bacterial cell wall and our results show that neither sub- nor supra-lethal amoxicillin or other bactericidal antibiotics cause intracellular superoxide formation in *E. faecalis* (Fig. 3f). How sublethal concentrations of erythromycin and chloramphenicol cause superoxide levels to increase could relate to the cell stress caused by inhibition of translation or by mistranslation, with the proteotoxic stress leading to increased reductive metabolism and thus increases in superoxide. The mechanism of erythromycin-induced superoxide production awaits further study.

**Fig. 7:**
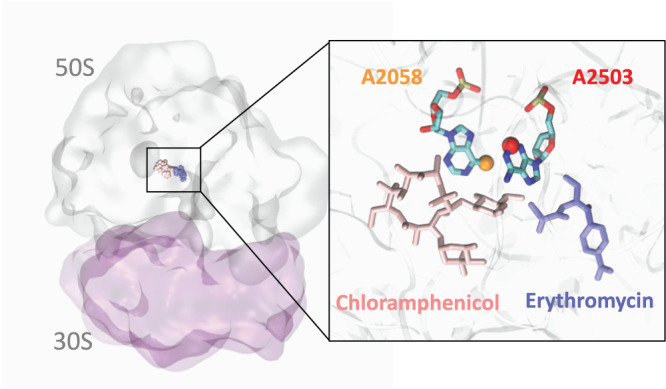
Structure of the ribosome showing superimposed binding sites for erythromycin, chloramphenicol, and positions of modified nucleotides A2503 and A2058. Figure includes erythromycin (blue), chloramphenicol (pink), the A2503 modified with m^2^A (red ball) by RlmN, and the A2058 site modified with Erm-mediated m^6^A (yellow ball).

How does RlmN-catalyzed m^2^A play a role in the phenotypic changes caused by loss of RlmN or exposure to superoxide? Two possibilities come to mind based on RlmN activity on both rRNA and tRNA. From the rRNA perspective, m^2^A might facilitate the ribosome stalling that leads to ErmBL nascent peptide activation of ErmB expression^31^. RlmN catalyzes m^2^A formation at A2503 of 23 S rRNA, a conserved nucleotide that resides in the peptidyl transferase center (PTC) of the ribosome near the entrance to the exit channel for the nascent polypeptide (Fig. 7) and is involved in fine-tuning ribosome–nascent peptide interactions, relaying the stalling signal to the PTC^32^. A2503 is very close spatially to the erythromycin binding site^32^, to the ErmBL nascent peptide that activates ErmB expression upon antibiotic binding^33^, and to the A2058 that is modified with m^6^A by ErmB to prevent antibiotic binding (Fig. 7). Though speculative, one hypothesis is that loss of RlmN activity reduces m^2^A2503 and thus facilitates ErmBL-induced activation of ErmB synthesis.

From the tRNA perspective, RlmN is one of only two methyltransferases known to modify both rRNA and tRNA^11^. In *E. coli*, m^2^A37 occurs in six tRNAs: tRNA^Arg^ICG, tRNA^Asp^QUC, tRNA^Gln^cmnm^5^sUUG, tRNA^Gln^CUG, tRNA^Glu^mnm^5^s^2^UUC, and tRNA^His^QUG^34^. Modifications at position 37 are important for maintaining the reading frame^35^, while loss of RlmN increases stop codon readthrough^36^. The latter is likely not relevant for synthesis of selenoproteins in *E. faecalis* since there are no apparent genes encoding selenoproteins in the *E. faecalis* genome^37^. Further studies are underway to determine if *E. faecalis* uses tRNA reprogramming and codon-biased translation to regulate expression of stress response genes as observed in mycobacteria^7^.

Finally, our results raise the question of the uniqueness of RlmN’s sensitivity to inactivation by superoxide, compared to other Fe-S cluster proteins in *E. faecalis* and other organisms. *E. faecalis* is unusual among human commensal and pathogenic bacteria in lacking many Fe-S cluster proteins, such as fumarase, aconitase, isocitrate dehydrogenase, and succinate dehydrogenase in the tricarboxylic acid cycle^38^. This lack of a tricarboxylate cycle is shared by *Listeria monocytogenes*, which also shares with *E. faecalis* a lack of ROS formation when exposed to bactericidal antibiotics^39^. Other Fe-S cluster proteins absent in *E. faecalis* include the GrxD iron transport regulator, 1 of 3 systems for Fe-S cluster biogenesis (NIF, ISC, and SUF; only SUF is present in *E. faecalis*^40^), and MiaB. The latter is corroborated by our inability to detect m^2^si^6^A in the presence of i^6^A (Fig. 1d). However, *E. faecalis* possesses several Fe-S cluster proteins, including QueE and QueG involved in queuosine (Q) biosynthesis^41^. Clearly more work is needed to determine the generality of ROS-sensitive Fe-S cluster RNA-modifying proteins in other organisms and homologous epitranscriptome-mediated signaling networks. It is certainly possible that RlmN is uniquely sensitive as a potential redox signaling node in *E. faecalis*. However, in addition to MiaB, there are other Fe-S cluster-containing RNA-modifying enzymes^42^ that, like RlmN, may serve as redox responsive regulators. A systematic analysis of ROS-sensitive RNA modifications in different microbes would advance our understanding of oxidative stress response networks in bacteria.

In conclusion, we showed that RlmN activity is not only strongly dependent upon superoxide levels but also regulates levels of SodA. In *E. faecalis* and other bacteria, SodA promotes antibiotic tolerance^30^ and facilitates survival in macrophages^23^. In all, RlmN, widely distributed across bacteria genera^43^, may serve as a redox switch relaying redox sensing to both the rRNA and tRNA epitranscriptome for direct modulation of translation for protective oxidative stress response.

## Methods

### Bacteria strains, plasmids, and growth conditions

*Enterococcus faecalis* strains OG1RF and V583 are grown in tryptic soy broth (TSB) or plated on tryptic soy agar under aerobic conditions at 37 °C. All mutant strains are derivatives of OG1RF. The *rlmN* knockout in OG1RF (Δ*rlmN*) was generated by an in-frame deletion of *rlmN* from OG1RF by allelic replacement using vector pGCP213^22^. The 250 bp regions upstream and downstream of the coding sequence of *rlmN* was amplified from OG1RF genomic DNA and stitched together by overlapping PCR and inserted into the XhoI/KpnI sites of pGCP213 (Supplementary Tables 5 and 6). This plasmid was transformed into OG1RF and introduced into the specific sites on the chromosome of the parental strain by recombinase-mediated gene replacement.

RlmN over-expressor, OG1RFp*rlmN*, was generated by the introduction of the gene coding for RlmN into the plasmid pGCP123 under the constitutive sortase promoter^22^. The coding sequence for RlmN was PCR amplified with a His6 tag at the C-terminus and inserted into the XhoI/NotI sites of pGCP123 under a Sortase A promoter (Supplementary Tables 5, 6). OG1RFp*Empty* is OG1RF carrying the plasmid pGCP123, but without introduction of the gene encoding RlmN. All the plasmids used in this study are listed in Supplementary Table 5. All the primers for cloning and RT-qPCR for making the mutant strains are listed in Supplementary Tables 6 and 7. All plasmid constructions were verified by Sanger sequencing. MICs used for OG1RF are 1 µg/mL erythromycin, 1 µg/mL spiramycin, 4 µg/mL chloramphenicol, 64 µg/mL kanamycin, 128 µg/mL streptomycin, 1 µg/mL ampicillin, 0.5 µg/mL tetracycline and 1 µg/mL ciprofloxacin. MICs used for V583 are >1024 µg/mL erythromycin, 8 µg/mL chloramphenicol, 256 µg/mL gentamicin, 1 µg/mL ampicillin, and 1 µg/mL ciprofloxacin. OG1RFp*rlmN* and OG1RFp*Empty* are grown in 500 µg/mL kanamycin to maintain the pGCP123 plasmid.

### RNA isolation and purification

An overnight culture was diluted 1:20 fold and then grown at 37 °C to reach mid-log phase. This mid-log phase culture was then diluted 1:20 into media containing sublethal concentrations of antibiotics and grown at 37 °C with shaking at 180 rpm. The cultures are harvested at an optical density (OD_600_) of ~0.6–0.8 after 4–5 doublings by centrifugation at 4000 × *g* for 10 min at 4 °C. RNA was extracted and purified following Hia et al.^44^. Briefly, 100 mL of bacteria culture was lysed in the presence of phenol:chloroform:isoamyl alcohol and 100 mM sodium acetate pH 5.0 by bead-beating with 0.1-mm zirconia-silica beads using Qiagen TissueLyser II for 12 min at 30 Hz. Large and small RNA species were differentially recovered using the PureLink miRNA Isolation Kit (Invitrogen) with 35% ethanol and 70% ethanol respectively. 23 S and 16 S rRNA are separately isolated to purity from the large RNA fraction following HPLC using the Bio SEC-5 column (Agilent; 7.8 mm, length: 300 mm, particle size: 5 μm, pore size: 1000 Å); and tRNA was isolated to purity from the small RNA fraction following HPLC on the Bio SEC-3 column (Agilent; 7.8 mm, length: 300 mm, particle size: 5 μm, pore size: 300 Å). All separations were run with 100 mM ammonium acetate, at 60 °C with a flow rate of 0.5 mL/min. The elution profiles for all samples are shown in Supplementary Fig. 1, which reveals highly purified samples of high-quality 16 S and 23 S rRNAs and tRNAs. HPLC fractions containing the target RNA populations were combined, concentrated, and subjected to buffer exchange into 10 mM ammonium acetate using 3000 Da cutoff size exclusion filters (Millipore). RNA was quantified by NanoDrop spectroscopy.

### Quantification of ribonucleosides by chromatography-coupled tandem mass spectrometry

For quantification of ribonucleosides, RNA (5 µg) was enzymatically hydrolyzed for 4 h at 37 °C with benzonase (99% purity, Novagen 70664), bacterial phosphatase (ThermoFisher 18011015) and phosphodiesterase I (Sigma P3243), in the presence of magnesium chloride, antioxidants and deaminase inhibitors including desferroxamine (Sigma D9533), butylated hydroxyltoluene (Sigma W218405), pentostatin (Sigma SML0508), tetrahydrouridine (Calbiochem 584222), and internal standard [^15^N]_5_-deoxyadenosine. Samples were cleaned up using a 10 kDa cutoff filter (Nanosep). The ribonucleoside mixtures were resolved on a Hypersil C18 analytical column (2.1 × 100 mm, 1.8 mm; Agilent) mounted on an Agilent 1290 HPLC system and linked to an Agilent 6490 triple-quadrupole mass spectrometer using multiple reaction monitoring in positive-ion mode. ESI parameters used were gas temperature 80 °C; gas flow 11 L/min; nebulizer pressure 20 psi; sheath gas temperature 300 °C; capillary voltage 1800 V; nozzle voltage 2000 V.

The ribonucleosides were identified using the retention times of standards and MS/MS mass transitions involving loss of either ribose (136 *m/z*) or 2’-*O*-methyl-ribose (146 *m/z*) (Supplementary Table 1). For relative quantitation of modifications among the same batch of samples, the signal intensity is normalized against the combined intensity of the four canonical ribonucleosides to correct for variation in RNA quantities. Spectral signals are also normalized against spiked internal standard ([^15^N]_5_−2’-deoxyadenosine) to adjust for variations in instrument sensitivity. For absolute quantification of m^2^A and adenosine, a series of concentrations of nucleoside standards for m^2^A and adenosine were run with every batch of samples to obtain standard calibration curves. The concentrations of nucleosides were then obtained by fitting the signal intensities onto the calibration curves, and these were then used to obtain the molar ratio of m^2^A/A.

Absolute quantification of m^2^A levels in rRNA and tRNA using calibration curves for adenosine and m^2^A reveals that m^2^A is present in 23 S rRNA in a ratio of approximately 0.00025 m^2^A per adenosine and at a 2.5-times higher ratio of 0.001 in tRNA. The level of reduction in m^2^A is identical between rRNA and tRNA, which decreases by ten-fold between untreated *E. faecalis* and cultures grown in 100-200 µg/mL of erythromycin (Fig. 1c, d). We approximated the abundance of m^2^A in rRNA and tRNA based on 23 S rRNA at 2904 nucleotides long and tRNA typically 76-90 nucleotides long, which equates to ~1m^2^A in 3.6 23 S rRNAs and ~1 in every 60 tRNAs in untreated V583 grown aerobically.

### Measurement of *rlmN* mRNA levels

Total RNA (2 µg) was subjected to DNA removal using the TURBO DNA-free kit (Ambion, Life Technologies) following manufacturer’s protocol. 600 ng in 15 µL was used for reverse transcription using the iScript cDNA synthesis kit (Bio-Rad, Hercules, CA, USA). The reverse transcription program was run as follows: 25 °C for 5 min, 42 °C for 30 min, and 85 °C for 5 min, followed by a cooling step at 4 °C. Two-step real-time quantitative polymerase chain reaction (qPCR) was then performed using the BlitzAmp qPCR mastermix (MiRxes, Singapore). Primer sequences can be found in Supplementary Table 8. The qPCR program was run as follows: 95 °C for 5 min followed by 40 cycles of denaturation at 95 °C for 10 s and annealing/extension at 60 °C for 30 s on a Bio-Rad CFX384 real-time PCR instrument and analyzed using the CFX manager 3.1. A melting curve analysis consisting of 0.5 °C increments from 65 to 95 °C was performed for all reactions to ascertain the specificity of the primers. RpoA served as an internal loading control.

### Measurement of RlmN protein levels

Since we could not detect RlmN in the TMT quantitative proteomics analyses, we performed targeted proteomics to quantify RlmN. Bacteria pellets from 10 mL of log phase culture of OG1RF and V583 were resuspended in 250 µL of 50 mM Hepes pH 8, 8 M urea, 1 mM DTT, and homogenized by bead-beating followed by clarification by centrifugation at 16,000 × *g* for 30 min at 4 °C. Following quantification of total protein in the supernatant using the bicinchoninic acid protein assay (ThermoFisher Scientific), 50 µg of protein was mixed with SDS-PAGE loading dye and separated on a 14% SDS-PAGE gel. Following staining and destaining, a gel slice corresponding to 30–45 kDa, encompassing target protein RlmN 40.9 kDa and reference proteins RpoA 35.05 kda and Gap2 35.77 kDa, was excised and cut into 1–2 mm pieces (Supplementary Fig. 4). The gel pieces were destained, reduced and alkylated followed by overnight trypsin digestion and peptide extraction following manufacturer’s instructions (In-Gel Tryptic Digestion kit, ThermoFisher Scientific). Extracted peptides were vacuum dried and redissolved in 2% acetonitrile in 0.1% formic acid in water.

Targeted quantification of RlmN was then achieved by first using Skyline (http://proteome.gs.washington.edu/software/skyline) to identify precursor peptides and transitions for RlmN and reference proteins RpoA and Gap2 (Supplementary Table 8). We analyzed the protein sequences of OG1RF RlmN, RpoA, and Gap2 against a background of the OG1RF proteome, looking for peptides 8–14 amino acids long, with the following Skyline settings: (1) trypsin cleavage; (2) minimum length 7 amino acids, maximum length of 25 amino acids, excluding 25 N-terminal amino acids; (3) excluding peptides containing Cys, Met, His, NXT/NXS, RP/KP; (4) including potential structural modifications (e.g., carbamidomethyl); and (5) maximum variable modifications at 3 and maximum neutral losses at 1. For RlmN, Skyline identified 3 target peptides: (1) K.QVIVQEAQDGTVK.Y [67, 79], (2) K.YLFELPDK.N [80, 87], and (3) K.GLAIGAR.H [184, 190], with peptides #1 and #2 selected. For Gap2, Skyline identified 5 target peptides: (1) K.YDTTQGR.F [46, 52], (2) K.AIGLVIPELNGK.L [215, 226], (3) K.LDGAAQR.V [227, 233], (4) R.VPVATGSLTELVTVLDK.E [234, 250], and (5) R.TLEYFANL.- [325, 332], with peptide #2 selected. For RpoA, Skyline identified 2 target peptides: (1) R.EDVTQIILNIK.G [70, 80] and (2) K.LYAEEEK.T [86, 92], with peptide #1 selected. Selected peptides were synthesized at 90% purity and analyzed by LC-MS/MS on a Hypersil C18 analytical column (2.1 × 100 mm, 1.8 mm; Agilent) mounted on an Agilent 1290 infinity LC system coupled to an Agilent 6490 QQQ spectrometer in positive-ion mode. Agilent Automated MRM Method Optimizer for Peptides was used to optimize collision energies and fragmentation voltages for their MRM transitions and peptides were used at a concentration of 10 µg/mL for determination of retention times. Reversed-phase chromatography was performed with a fixed flow rate of 0.25 mL/min with a gradient of water and acetonitrile (solvent B) acidified with 0.1% (v/v) formic acid. Gradients used were as follow: 0–29% solvent B from 0–29 min, 29–90% from 29–30 min, 90% for 38 min, 90 to 0 % from 38 to 39 min, and 0% for 45 min. Source conditions: gas temperature 325 °C, gas flow 10 L/min, nebulizer 32 psi, sheath gas temperature 300 °C, sheath gas flow 11 L/min, capillary 2000 V, charging 500 V. Columns were incubated at 40 °C. The top two precursor peptides by peak area and number of transitions (minimum 2) were selected as qualification and quantification ions. Following the definition of the analytical parameters for the synthetic peptides, the peptides extracted from the gel slices were then analyzed on the same LC-MS/MS system. Signal intensities for each peptide were normalized to the total signal intensity for the LC-MS/MS run to account for peptide loading differences and the fold-change data were calculated by dividing normalized signal intensities for antibiotic-treated samples by those in control samples.

### Flow cytometry assays

CellROX Green (ThermoFisher) is a proprietary oxidation-sensitive dye whose fluorescence at 500–550 nm after excitation at 488 nm increases substantially on oxidation in the presence of dsDNA. Cellrox green reacts to hydroxyl radical and superoxide but not hydrogen peroxide^15^. Log-phase cultures were diluted to OD_600_ of 0.1 in 10% TSB in the presence of menadione or antibiotics and incubated for 30 min at 37 °C followed by the addition of CellROX green (final 0.5 µM) for a further 30 mins at 37 °C in the dark with shaking at 180 rpm. Samples were analyzed using a HTS autosampler system on the Attune Nxt v4.2.0 (ThermoFisher Scientific) and the flow rate was set to 25 µL/min with a 30 µL injection volume. Samples were detected with a 530/30 nm band-pass emission and recording 50,000 events in the bacterial gate. Gating was set using unstained samples for the bacterial population by forward-scatter (FSC; correlates with cell size) and side-scatter (SSC; correlates with cell internal granularity) of light to determine background fluorescence. Data were analyzed with the Attune Nxt software.

### Proteomics

Fresh mid-log phase cultures (OD_600_ of 0.6) were diluted 1:20 into TSB media with or without treatment and grown at 37 °C with shaking at 180 rpm. The cultures are harvested at an optical density (OD_600_) of ~0.6–0.8 after 4–5 doublings by centrifugation at 4000 × *g* for 10 min at 4 °C. Bacterial pellets were resuspended in 250 µL of 50 mM Hepes pH 8, 8 M urea, 1 mM DTT, and homogenized by bead-beating followed by clarification by centrifugation at 16,000 × *g* for 30 min at 4 °C. Following protein quantification in the supernatant using the bicinchoninic acid protein assay (ThermoFisher Scientific), 200 µg of protein was digested with trypsin after being reduced with DTT and alkylated with iodoacetamide. After washing 3 times with 0.5 M TEAB followed by fractionation using the 10 kDa ultrafiltration system, ~100 μg of digested peptides from each group, including two biological replicates, was labeled using the six-plex TMT isobaric and isotopic mass-tagging kit (ThermoFisher Scientific), which was performed according to manufacturer’s instructions.

Peptides were separated by reverse phase HPLC (Thermo Easy nLC1000) using a precolumn (made in house, 6 cm of 10 µm C18) and a self-pack 5 µm tip analytical column (15 cm of 5 µm C18, New Objective) over a 150 min gradient before nanoelectrospray using a QExactive mass spectrometer (ThermoFisher). The mass spectrometer was operated in a data-dependent mode. The parameters for the full scan MS were as follow: resolution of 70,000 across 350–2000 *m/z*, AGC 3e6, and maximum IT 50 ms. The full MS scan was followed by MS/MS for the top 15 precursor ions in each cycle with a NCE of 28 and dynamic exclusion of 30 s. Raw mass spectral data files (.raw) were searched using Proteome Discoverer (ThermoFisher) and Sequest. Search parameters were as follows: 10 ppm mass tolerance for precursor ions; 0.8 Da for fragment ion mass tolerance; 2 missed cleavages of trypsin; fixed modification was carbamidomethylation of cysteine, and N-term and Lysine TMT-label; variable modifications were methionine oxidation and serine, threonine and tyrosine phosphorylation. Only peptides with a Scorer score ≥2, reporter channel intensity >500 and an isolation interference ≤30 were included in the data analysis. Proteomics data are presented in Supplementary Data 1.

### Bactericidal activity analysis

A log phase culture of OG1RF was diluted to a final concentration of 10^8^ CFU/mL (OD_600_ ~ 0.1) in Tryptic Soy Broth in the presence of antibiotics at 37 °C with shaking. Aliquots were drawn from each respective tube at various time points and serially diluted until 10^−8^-fold. An aliquot (2.5 µL) of each dilution was spotted onto TSB agar and incubated at 37 °C overnight, with colonies counted 24 h after spotting.

### Determination of minimal inhibitory concentrations (MIC) of antibiotics

Two-fold serial dilutions of antibiotics in TSB were performed in separate rows of a polystyrene 96-well plate (Corning) with each plate containing an inoculum of respective bacteria. The inoculum was a 1:500 dilution from a culture at log phase (OD_600_ = 0.5) grown at 37 °C. The plate was incubated with shaking at 37 °C and the optical density of each well was measured at a wavelength of 600 nm (BIOTEK, Synergy 4). The MIC values were taken as the lowest concentration for which no growth was discernible (<0.05 OD_600_) after 24 h. All tests were performed three times independently with two samples in each test. MIC data are presented in Supplementary Table 2.

### Antibiotic killing assays

The OG1RF strains were cultured aerobically in TSB 37 °C for approximately 16 h with shaking (180 rpm) followed by 1:20 dilution and cultured to mid-log growth. Strains OG1RFp*Empty* and OG1RFp*rlmN* harboring pGCP123 plasmids were grown in 500 µg/mL kanamycin. The mid-log culture was diluted to OD_600_ ~ 0.06 (~5 × 10^7^ cfu/mL). An aliquot was plated to enumerate the colony-forming units (cfu) (Time 0) before the addition of antibiotics with final concentrations at 20 µg/mL ciprofloxacin and 20 µg/mL ampicillin. An aliquot was removed at the indicated time points and washed with sterile PBS. The cells were serially diluted and plated on tryptic soy agar to enumerate the survivors.

### Statistical analysis

Statistical significance was assessed using appropriate tests using Prism 8 (GraphPad) software, detailed in their respective figure legends. Asterisks indicate the level of statistical significance: *P < 0.05, **P < 0.01, ***P < 0.001, and ****P < 0.0001. *P* values < 0.05 were considered significant. Experiments were repeated at least three times.

### Reporting summary

Further information on research design is available in the Nature Portfolio Reporting Summary linked to this article.

## Supplementary information

Supplementary Information

Reporting Summary

## Data Availability

The data supporting the findings of this study are available from the corresponding authors upon reasonable request. The mass spectrometry proteomics data have been deposited to the ProteomeXchange Consortium via the PRIDE partner repository with the accession code PXD038178. The source data used to generate plots are provided as a Source Data file. Source data are provided with this paper.

## Source data

Source Data
