# Supplementary material for: An RNA modification enzyme directly senses reactive oxygen species for translational regulation in Enterococcus faecalis
Source: Nat Commun. 2023 Jul 11;14:4093. doi: 10.1038/s41467-023-39790-x (PMC10336011; doi:10.1038/s41467-023-39790-x)
Supplement: Supplementary file 1 — Supplementary Information [file 41467_2023_39790_MOESM1_ESM.pdf]

**Supplementary Information**

**"An RNA modification enzyme directly senses reactive oxygen species for translational regulation in *Enterococcus faecalis*" by Lee *et al.***

**Table of contents**


- **Supplementary figures**
  - **Supplementary Figure 1**. Size-exclusion HPLC traces showing on SEC 5, fractionation of the 23S and 16S rRNA peaks, and on SEC 3, fractionation of the 5S rRNA and tRNA peaks.
  - **Supplementary Figure 2**. LC-MS/MS extracted ion chromatograms of modified ribonucleosides analyzed in V583 23S and 16S rRNA and tRNA.
  - **Supplementary Figure 3.** Epitranscriptomic profiling of 23S and tRNA of OG1RF grown in the presence of sub-inhibitory concentrations of erythromycin.
  - **Supplementary Figure 4**. SDS-PAGE of whole cell lysates of V583 and OG1RF upon erythromycin treatment, for targeted proteomics of RlmN.
  - **Supplementary Figure 5**. Representative contour plots of CellROX Green-stained OG1RF treated with various antibiotics
  - **Supplementary Figure 6**. Mean FSC-H and SSC-H values for CellROX Green-stained OG1RF cells treated with various antibiotics

- **Supplementary tables**
  - **Supplementary Table 1**. Table of ribonucleoside to base ion mass transitions and transition times for monitored ribonucleoside modifications in V583.
  - **Supplementary Table 2.** Minimum inhibitory concentrations (MIC, µg/mL) of V583 and OG1RF WT and strains using a broth microdilution assay
  - **Supplementary Table 3.** Proteins significantly up- and down-regulated in the Δ*rlmN* mutant.
  - **Supplementary Table 4.** Proteins significantly up- and down-regulated in both the Δ*rlmN* mutant and following menadione treatment.
  - **Supplementary Table 5.** Plasmids used in this study
  - **Supplementary Table 6.** Cloning primers used in this study
  - **Supplementary Table 7.** Primers used for RT-qPCR
  - **Supplementary Table 8.** Peptides used for targeted protein mass spectrometry

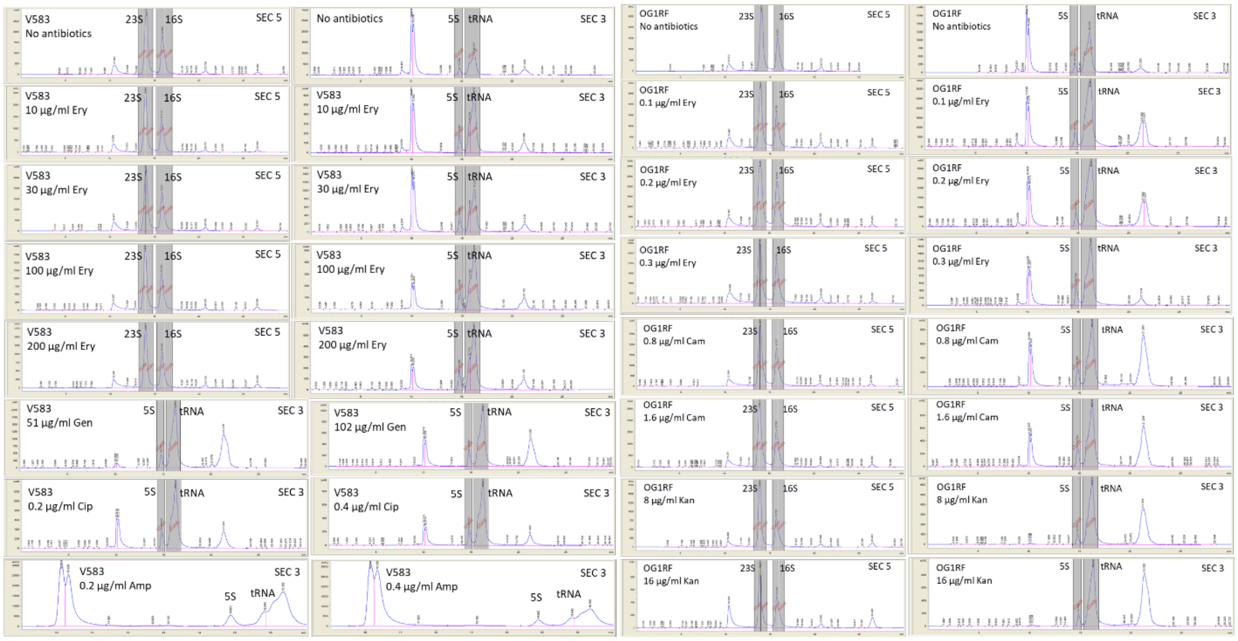


**Supplementary Figure 1.** Representative size-exclusion HPLC chromatograms for tRNA and rRNA purification attest to the quality and purity of the RNA samples. SEC 5: Bio SEC-5 column with 1000 Å pore size for purifying 23S and 16S rRNAs. SEC 3: Bio SEC-3 column with 300 Å pore size for purifying 5S rRNA and tRNA. Grey-shaded bars bracket the elution time window used to collect individual fractions. The differences in proportion of large rRNA in the SEC 3 profile (peak eluting before 5S rRNA) arises from carry-over from the first PureLink RNA purification column during differential ethanol precipitation. The peaks eluting after tRNA in the SEC 3 chromatograms represent small DNA/RNA fragments or other UV-absorbing contaminants. The two panels on the lower right (V583 0.2 and 0.4 µg/ml Amp, SEC3) represent zoom-ins of the SEC3 elution profile to show the quality of the RNA.

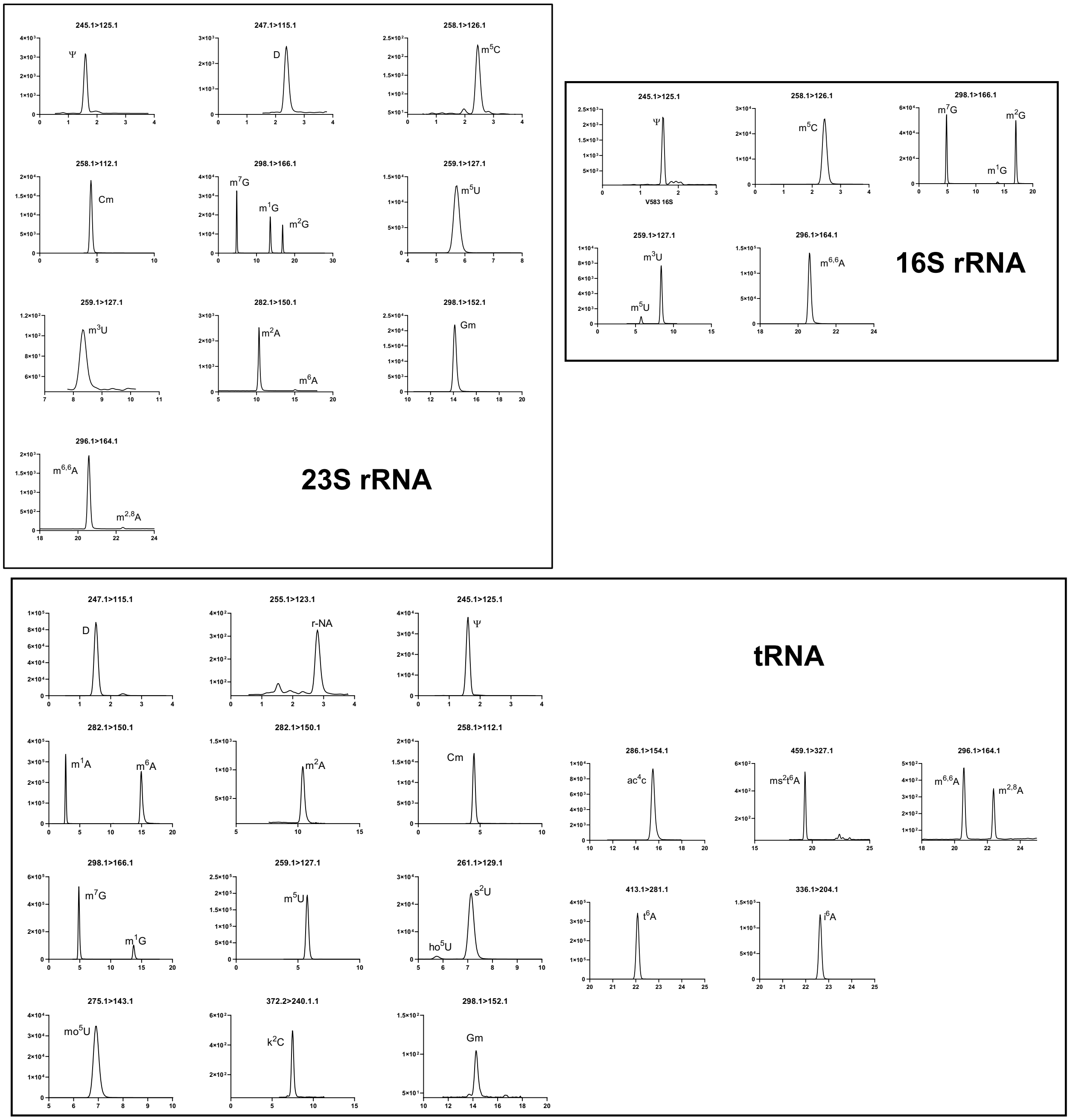


**Supplementary Figure 2.** LC-MS/MS extracted ion chromatograms of modified ribonucleosides analyzed in V583 23S and 16S rRNA and tRNA. The “X > Y” values denote collision-induced dissociation mass transitions involving the loss of either ribose (136 *m/z*) or 2’-*O*-methyl-ribose (146 *m/z*).

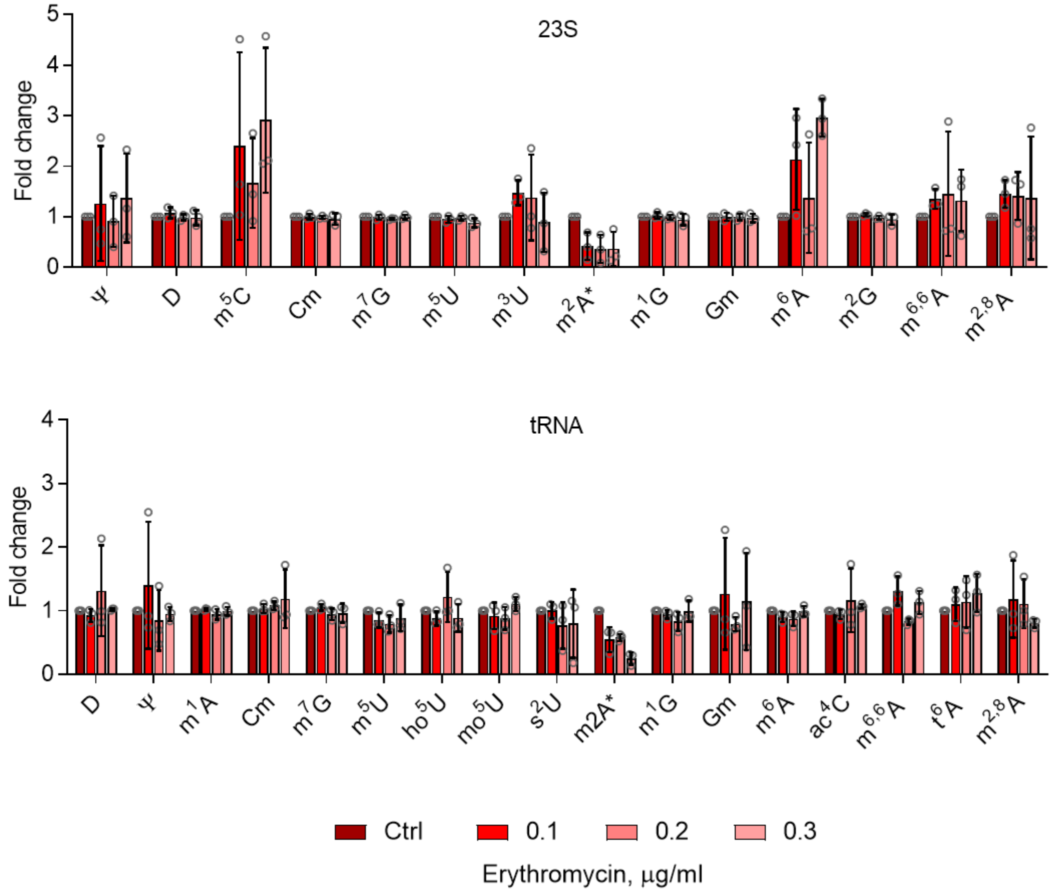


**Supplementary Figure 3.** Epitranscriptomic profiling of 23S and tRNA of OG1RF grown in the presence of sub-inhibitory concentrations of erythromycin. Changes in RNA modifications in OG1RF in erythromycin (0.1, 0.2 and 0.3 µg/ml) compared to untreated. Levels of modifications are shown as fold change to that within the untreated control. 23S rRNA (top panel) and tRNA (bottom panel). Modifications are arranged from left to right in ascending retention times. m2A is shown with an asterisk. Full names, precursor and product ion masses and retention times of RNA modifications can be found in Supplementary Table 1. All data are derived from three independent experiments (mean ± SD, $n = 3$). Source data are provided as a Source Data file.

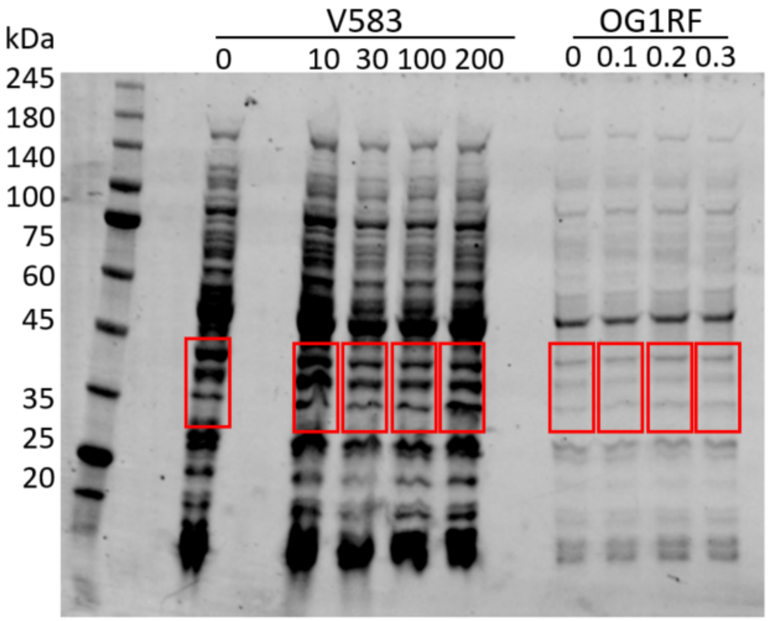


**Supplementary Figure 4**. In preparation for targeted proteomics quantification of RlmN, whole-cell lysates of V583 and OG1RF, exposed to erythromycin at the doses indicated above each lane (μg/mL), were resolved by SDS-PAGE. Red boxes demarcate the gel slice excised for in-gel trypsin digestion. The gel slices correspond to ~30-45 kDa to encompass target protein RlmN (40.9 kDa) and reference proteins RpoA (35.05 kDa) and Gap2 (35.77 kDa). Left lane: ExactPro Broad Range (5-245kDa) Prestained Protein Ladder (1st BASE). Similar gel images were obtained from three independent biological replicates. Related to Figure 3.

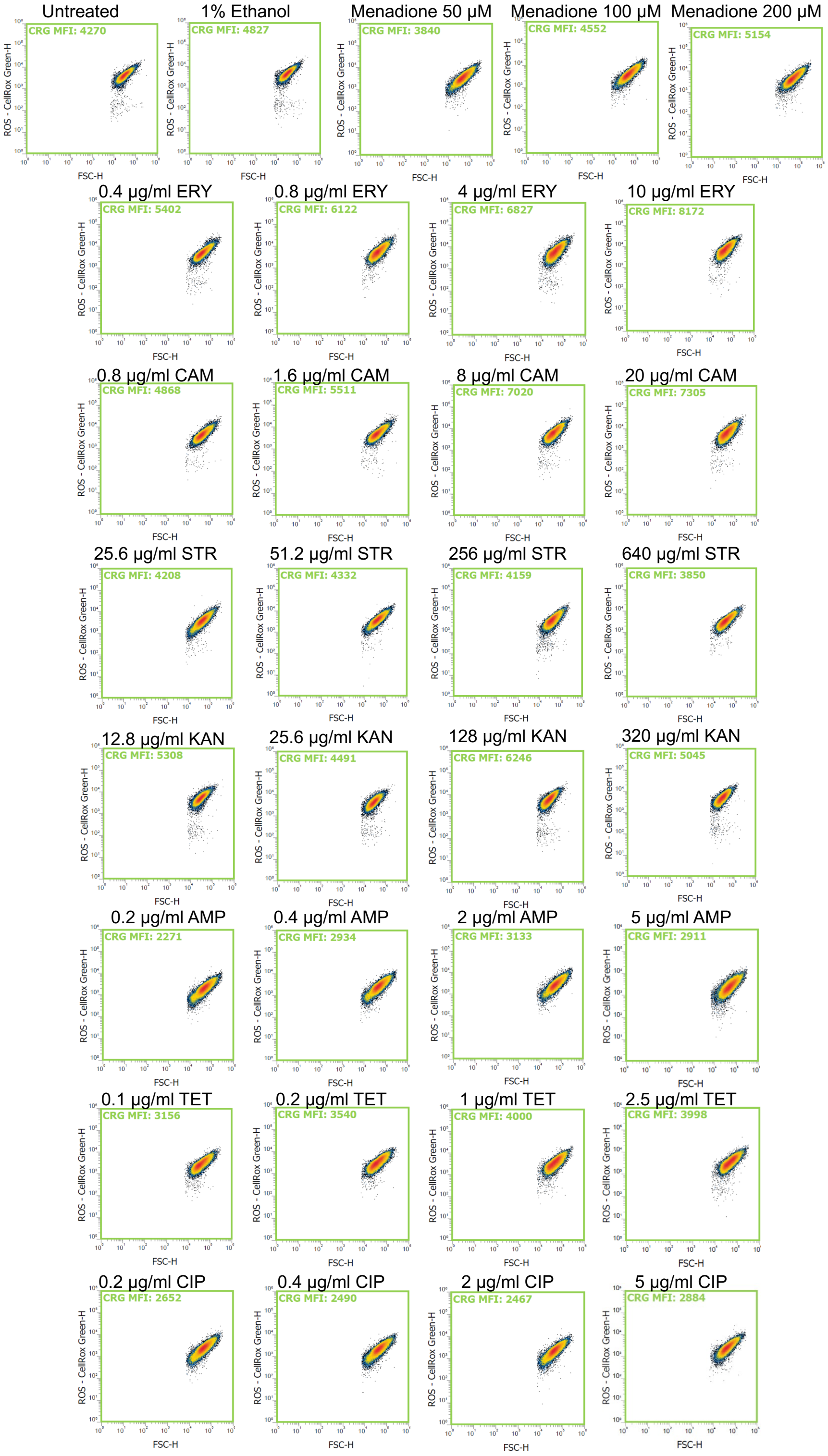


**Supplementary Figure 5**. Representative contour plots of CellROX Green-stained OG1RF treated with various antibiotics at indicated concentrations. ERY, erythromycin, CAM chloramphenicol, STR streptomycin, KAN kanamycin, AMP ampicillin, TET tetracycline, CIP ciprofloxacin.

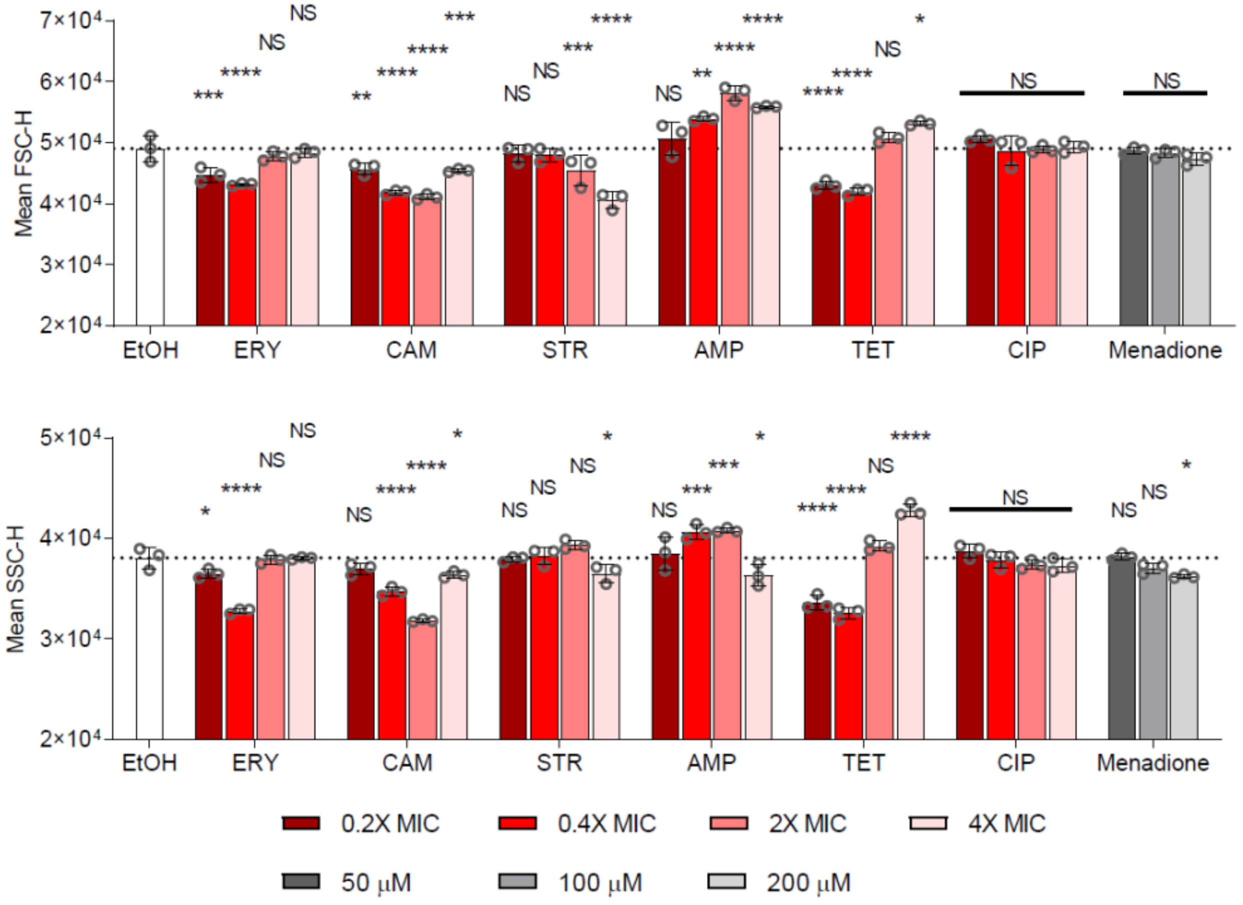


**Supplementary Figure 6**. Mean FSC-H (upper) and SSC-H (lower) values for CellROX Green-stained OG1RF cells treated with various antibiotics at the indicated concentrations. Data represent mean ± SD for three independent experiments. The observed changes in mean FSC-H and SSC-H were not consistent with the changes in mean fluorescence intensity in Figure 3F, confirming that the observed fluorescence intensity of CellROX Green was not significantly confounded by changes in bacterial size. Statistical analysis by two-way analysis of variance (ANOVA) with Dunnett's test versus EtOH: NS, not significant (P>0.05); P<0.05, P<0.005, P<0.0005 and P<0.0001 are denoted as *, **, *** and ****, respectively. FSC-H exact *p* values: ERY 0.2x MIC, 0.0006; ERY 0.4x MIC, <0.0001; CAM 0.2x MIC, 0.0063; CAM 0.4x MIC, <0.0001; CAM 2x MIC, <0.0001; CAM 4x MIC, 0.0031; STR 2x MIC, 0.0028; STR 4x MIC, <0.0001; AMP 0.4x MIC, 0.0227, AMP 2x MIC, <0.0001; AMP 4x MIC, 0.0002; TET 0.2x MIC, <0.0001; TET 0.4x MIC, <0.0001. SSC-H exact *p* values: ERY 0.2x MIC, 0.0450, ERY 0.4x MIC, <0.0001; CAM 0.4x MIC, <0.0001; CAM 2x MIC, <0.0001; CAM 4x MIC; 0.0277; STR 4x MIC, 0.0419; AMP 0.4x MIC, 0.0003; AMP 2x MIC, 0.0001; AMP 4x MIC, 0.0229; TET 0.2x MIC <0.0001; TET 0.4x MIC, <0.0001; TET 4x MIC, <0.0001; Menadione 200 μM, 0.0128.

**Supplementary Table 1.** Table of ribonucleoside to base ion mass transitions and transition times for monitored ribonucleoside modifications in V583. Related to Figure S1.

| | Modified ribonucleosides | Abbrev | Retention Time (min) | Precursor ion (*m/z*) | Product ion (*m/z*) | Neutral Loss (amu) |
|---|---|---|---|---|---|---|
| | | | **23S ribonucleosides** | | | |
| 1 | Pseudouridine | Ψ | 1.6 | 245.1 | 125.1 | 120 |
| 2 | Dihydrouridine | D | 2.3 | 247.1 | 115.1 | 132 |
| 3 | 5-Methylcytidine | $m^5C$ | 2.4 | 258.1 | 126.1 | 132 |
| 4 | 2'-O-Methylcytidine | Cm | 4.5 | 258.1 | 112.1 | 146 |
| 5 | 7-Methylguanosine | $m^7G$ | 4.8 | 298.1 | 166.1 | 132 |
| 6 | 5-Methyluridine | $m^5U$ | 5.7 | 259.1 | 127.1 | 132 |
| 7 | 3-Methyluridine | $m^3U$ | 8.3 | 259.1 | 127.1 | 132 |
| 8 | 2-Methyladenosine | $m^2A$ | 10.5 | 282.1 | 150.1 | 132 |
| 9 | 1-Methylguanosine | $m^1G$ | 13.7 | 298.1 | 166.1 | 132 |
| 10 | 2'-O-Methylguanosine | Gm | 14.3 | 298.1 | 152.1 | 146 |
| 11 | 6-Methyladenosine | $m^6A$ | 15.1 | 282.1 | 150.1 | 132 |
| 12 | 2-Methylguanosine | $m^2G$ | 16.9 | 298.1 | 166.1 | 132 |
| 13 | $N^6$, $N^6$-Dimethyladenosine | $m^{6,6}A$ | 20.6 | 296.1 | 164.1 | 132 |
| 14 | 2,8-Dimethyladenosine | $m^{2,8}A$ | 22.3 | 296.1 | 164.1 | 132 |
| | | | **16S ribonucleosides** | | | |
| 1 | Pseudouridine | Ψ | 1.6 | 245.1 | 125.1 | 120 |
| 2 | 5-methylcytidine | $m^5C$ | 2.4 | 258.1 | 126.1 | 132 |
| 3 | 7-Methylguanosine | $m^7G$ | 4.8 | 298.1 | 166.1 | 132 |
| 4 | 5-Methyluridine | $m^5U$ | 5.7 | 259.1 | 127.1 | 132 |
| 5 | 3-Methyluridine | $m^3U$ | 8.3 | 259.1 | 127.1 | 132 |
| 6 | 1-Methylguanosine | $m^1G$ | 13.7 | 298.1 | 166.1 | 132 |
| 7 | 2-Methylguanosine | $m^2G$ | 16.9 | 298.1 | 166.1 | 132 |
| 8 | $N^6$, $N^6$-Dimethyladenosine | $m^{6,6}A$ | 20.6 | 296.1 | 164.1 | 132 |
| | | | **tRNA ribonucleosides** | | | |
| 1 | Dihydrouridine | D | 1.5 | 247.1 | 115.1 | 132 |
| 2 | N-Ribosylnicotinamide | r-NA | 2.8 | 255.1 | 123.1 | 132 |
| 3 | Pseudouridine | Ψ | 1.6 | 245.1 | 125.1 | 120 |
| 4 | 1-Methyladenosine | $m^1A$ | 2.7 | 282.1 | 150.1 | 132 |
| 5 | 2'-O-Methylcytidine | Cm | 4.5 | 258.1 | 112.1 | 146 |
| 6 | 7-Methylguanosine | $m^7G$ | 4.9 | 298.1 | 166.1 | 132 |
| 7 | 3-Methyluridine | $m^5U$ | 5.8 | 259.1 | 127.1 | 132 |
| 8 | 5-hydroxyuridine | $ho^5U$ | 5.8 | 261.1 | 129.1 | 132 |
| 9 | 5-methoxyuridine | $mo^5U$ | 7.0 | 275.1 | 143.1 | 132 |
| 10 | 2-Thiouridine | $s^2U$ | 7.1 | 261.1 | 129.1 | 132 |
| 11 | 2-Lysidine | $k^2C$ | 7.5 | 372.2 | 240.1 | 132.1 |
| 12 | 2-Methyladenosine | $m^2A$ | 10.4 | 282.1 | 150.1 | 132 |
| 13 | 1-Methylguanosine | $m^1G$ | 13.7 | 298.1 | 166.1 | 132 |
| 15 | 2'-O-Methylguanosine | Gm | 14.3 | 298.1 | 152.1 | 146 |
| 16 | 6-Methyladenosine | $m^6A$ | 15.0 | 282.1 | 150.1 | 132 |
| 17 | $N^4$-acetylcytidine | $ac^4C$ | 15.5 | 286.1 | 154.1 | 132 |
| 18 | 2-Methylthio-$N^6$-threonylcarbamoyladenosine | $ms^2t^6A$ | 19.4 | 459.1 | 327.1 | 132 |
| 19 | $N^6$, $N^6$-Dimethyladenosine | $m^{6,6}A$ | 20.6 | 296.1 | 164.1 | 132 |
| 20 | $N^6$-Threonylcarbamoyl adenosine | $t^6A$ | 22.1 | 413.1 | 281.1 | 132 |
| 21 | 2,8-Dimethyladenosine | $m^{2,8}A$ | 22.4 | 296.1 | 164.1 | 132 |
| 22 | $N^6$-Isopentenyladenosine | $i^6A$ | 22.6 | 336.16 | 204.16 | 132 |

**Supplementary Table 2.** Minimum inhibitory concentrations (MIC, μg/mL) of V583 and OG1RF WT and strains using a broth microdilution assay.

| | Erythro-mycin | Chloram-phenicol | Tetra-cycline | Ampi-cillin | Cipro-floxacin | Genta-micin | Kana-mycin |
|---|---|---|---|---|---|---|---|
| **OG1RF WT** | 1 | 4 | 0.5 | 1 | 1 | 32 | 64 |
| **OG1RF Δ*rlmN*** | 1 | 64 | 0.5 | 1 | 1 | 32 | 64 |
| **V583** | >256 | 8 | 1 | 1 | 1 | 256 | >256 |
| **OG1RFp*Empty*** | 1 | 4 | 0.5 | 1 | 1 | 32 | >256 |
| **OG1RFp*rlmN*** | 1 | 4 | 0.5 | 1 | 1 | 32 | >256 |

**Supplementary Table 3.** Proteins significantly up- and down-regulated in the Δ*rlmN* mutant. Cut-off is set at 1 standard deviation, which amounts to a $\log_2$(fold-change) of ±0.443.

| **Locus** | **Protein Description** | **Molecular function** | **Δ*rlmN*** | |
|---|---|---|---|---|
| | | | **$\log_2$(FC)** | **$-\log_{10}$ (*p*-value)** |
| **INCREASED RELATIVE TO CONTROL** | | | | |
| OG1RF_10574 | D-Alanine--D-alanine ligase, Ddl | Cell wall formation, peptidoglycan biosynthesis | 0.577 | 1.699 |
| OG1RF_10348 | Superoxide dismutase, SodA | Metal ion binding; superoxide dismutase activity | 0.529 | 2.337 |
| **DECREASED RELATIVE TO CONTROL** | | | | |
| OG1RF_11526 | M4 family metallopeptidase coccolysin, gelatinase, GelE | Hydrolyzes azocoll, gelatin, and collagens | -0.522 | 1.412 |
| OG1RF_10742 | DEAD/DEAH box family ATP-dependent RNA helicase | Nucleic acid binding | -0.532 | 1.580 |
| OG1RF_10448 | Phosphocarrier protein HPr, PTS family porter, PtsI | Phosphoenolpyruvate-dependent sugar phosphotransferase system | -0.628 | 2.195 |
| OG1RF_10487 | WxL domain-containing protein | Surface cell wall-binding | -0.692 | 1.349 |
| OG1RF_10489 | WxL domain-containing protein | Surface cell wall-binding | -0.760 | 1.307 |
| OG1RF_10486 | WxL domain-containing protein | Surface cell wall-binding | -0.763 | 1.516 |
| OG1RF_10869 | Endocarditis and biofilm-associated pilus tip protein EbpA | Pilus biogenesis, biofilm formation | -0.844 | 1.456 |
| OG1RF_10155 | 30S Ribosomal protein S19, RpsS | Structural constituent of ribosome | -1.003 | 1.686 |
| OG1RF_12327 | Class A sortase, SrtA | Transpeptidase that anchors surface proteins to the cell wall | -1.315 | 1.702 |
| OG1RF_12485 | Alkaline phosphatase family protein, phosphatidylglycerol--membrane-oligosaccharide glycerophosphotransferase | Membrane biogenesis | -1.352 | 2.333 |
| OG1RF_10639 | ABC transporter ATP-binding protein, Opp1F | Membrane transport | -1.681 | 1.796 |

**Supplementary Table 4.** Proteins significantly up- and down-regulated in both the Δ*rlmN* mutant and following menadione treatment. Cut-off set at 1 standard deviation, which amounts to a $\log_2$(fold-change) of ±0.443 for Δ*rlmN* and ±0.639 for menadione treatment.

| Locus | Protein Description | Molecular function | Menadione treatment | | Δ*rlmN* | |
|---|---|---|---|---|---|---|
| | | | $Log_2$(FC) | $-Log_{10}$ (*p*-value) | $Log_2$(FC) | $-Log_{10}$ (*p*-value) |
| **INCREASED RELATIVE TO CONTROL** | | | | | | |
| OG1RF_10348 | Superoxide dismutase (SodA) | Metal ion binding; superoxide dismutase activity | 1.762 | 1.985 | 0.529 | 2.337 |
| **DECREASED RELATIVE TO CONTROL** | | | | | | |
| OG1RF_10869 | Endocarditis and biofilm-associated pilus tip protein EbpA | Pilus biogenesis, biofilm formation | -1.055 | 1.630 | -0.844 | 1.516 |
| OG1RF_10487 | WxL domain-containing protein | Surface cell wall-binding | -2.026 | 1.560 | -0.692 | 1.456 |
| OG1RF_10489 | WxL domain-containing protein | Surface cell wall-binding | -2.832 | 1.952 | -0.760 | 1.349 |
| OG1RF_10486 | WxL domain-containing protein | Surface cell wall-binding | -2.869 | 2.166 | -0.763 | 1.307 |

**Supplementary Table 5.** Plasmids used in this study

| Name | Selection Marker | Description | Reference |
|---|---|---|---|
| pGCP123 pSrtA | Kanamycin | Under constitutive Sortase A promoter | |
| pGCP213 | Erythromycin | | (Nielsen *et al.*, 2012) |
| pGCP123 pSrtA RlmN | Kanamycin | Overexpression of RlmN | This study |

**Supplementary Table 6.** Cloning primers used in this study.

| Name | Description | Method of cloning | Sequence 5'-3' |
| --- | --- | --- | --- |
| OG1RF Δ*rlmN* | *rlmN* knock out in OG1RF | Restriction digestion | F-XhoI-rlmn250-pGCP213: Catgc-ctcgag-AATTTCACTTTCTTGAAAAGATAACG RC1-rlmn-del- pGCP213: GAAATGAGGAAAAGAACGTAGTTAAAATCGGATCAGAAAGG F2-rlmn-del- pGCP213: CCGATTTTAACTACG-TTCTTTTCCTCATTTCTGCTATTAC RC-rlmn250-kpnI- pGCP213: gcagtggtaccTTAGATCAGCCAATGCAATTAGCTG |
| OG1RF p*rlmN* | RlmN Over-expression using a sortase A promoter in pGCP123 | Restriction digestion | F-XhoI-rlmn-PsrtA: Catgc-ctcgag-Atgcagaaagaatccatttatgg RC-rlmn-NotI-PsrtA: gcagtgcggccgcgtgatggtggtgatgatgttattggtttttgactttttctttT |

**Supplementary Table 7.** Primers used for RT-qPCR

| mRNA | Primers | Product length |
| --- | --- | --- |
| *rlmN* | CCACTCAAGTTGGCTGCAAT; CCAACCCACGTTCATCGAAA | 135 |
| | GCAAGAAGCGCAAGATGGTA; CAACAATCTCGCCAGCAGTT | 197 |
| *rpoA* | ACAGTGAAACCTGGTCGTGG; TCATCACGACGACCAACACG | 149 |

**Supplementary Table 8.** Peptides used for targeted protein mass spectrometry

| Proteins | Peptides | Precursor ion | Product ion | Retention time (min) | Synthesized purity and quantity | Y ion |
| --- | --- | --- | --- | --- | --- | --- |
| RlmN, Dual-specificity RNA methyl-transferase | QVIVQEAQDGTVK | 707.7 | 1074.5 | 13.8 | 90% Pure, 5~9 mg | y10 |
| | YLFELPDK | 513.089 | 359.3 | 24.3 | 90% Pure, 5~9 mg | y3 |
| RPOA, RNA polymerase subunit alpha | EDVTQIILNIK | 643.8 | 600.2 | 27.8 | 90% Pure, 5~9 mg | y5 |
| GAP2, Glyceraldehyde-3-phosphate dehydrogenase | AIGLVIPELNGK | 612.7 | 657.3 | 26.3 | 90% Pure, 5~9 mg | y6 |
