# Supplementary material for: An RNA modification enzyme directly senses reactive oxygen species for translational regulation in Enterococcus faecalis
Source: Nat Commun. 2023 Jul 11;14:4093. doi: 10.1038/s41467-023-39790-x (PMC10336011; doi:10.1038/s41467-023-39790-x)
Supplement: Supplementary file 2 — Reporting Summary [file 41467_2023_39790_MOESM2_ESM.pdf]

nature portfolio

Corresponding author(s): Peter Dedon

Last updated by author(s): Feb 25, 2023

Reporting Summary

Nature Portfolio wishes to improve the reproducibility of the work that we publish. This form provides structure for consistency and transparency in reporting. For further information on Nature Portfolio policies, see our Editorial Policies and the Editorial Policy Checklist.

Statistics

For all statistical analyses, confirm that the following items are present in the figure legend, table legend, main text, or Methods section.

n/a	Confirmed	
☐	☒	The exact sample size (*n*) for each experimental group/condition, given as a discrete number and unit of measurement
☐	☒	A statement on whether measurements were taken from distinct samples or whether the same sample was measured repeatedly
☐	☒	The statistical test(s) used AND whether they are one- or two-sided<br>*Only common tests should be described solely by name; describe more complex techniques in the Methods section.*
☒	☐	A description of all covariates tested
☒	☐	A description of any assumptions or corrections, such as tests of normality and adjustment for multiple comparisons
☒	☐	A full description of the statistical parameters including central tendency (e.g. means) or other basic estimates (e.g. regression coefficient) AND variation (e.g. standard deviation) or associated estimates of uncertainty (e.g. confidence intervals)
☒	☐	For null hypothesis testing, the test statistic (e.g. *F*, *t*, *r*) with confidence intervals, effect sizes, degrees of freedom and *P* value noted<br>*Give P values as exact values whenever suitable.*
☒	☐	For Bayesian analysis, information on the choice of priors and Markov chain Monte Carlo settings
☒	☐	For hierarchical and complex designs, identification of the appropriate level for tests and full reporting of outcomes
☒	☐	Estimates of effect sizes (e.g. Cohen's *d*, Pearson's *r*), indicating how they were calculated

Our web collection on statistics for biologists contains articles on many of the points above.

Software and code

Policy information about availability of computer code

Data collection	RNA modifications: MassHunter Workstation Data Acquisition;<br>qPCR: CFX Maestro Software (Bio-Rad);<br>Flow cytometry: Attune Nxt (Thermo Fisher Scientific);<br>Bacterial growth assays: BIOTEK Gen5 (Agilent)
Data analysis	RNA modifications: MassHunter Workstation Quantitative Analysis and Qualitative Analysis software (B.06);<br>qPCR: CFX Maestro Software (Bio-Rad);<br>Flow cytometry: Attune Nxt (Thermo Fisher Scientific);<br>Targeted peptide mass spec: Skyline (MacCoss Lab);<br>Proteomics: Proteome Discoverer (ThermoFisher), Sequest;<br>General data analysis: Prism 8 (Graphpad), Excel 2013 (Microsoft);<br>Sanger sequencing: BioEdit 7.2

For manuscripts utilizing custom algorithms or software that are central to the research but not yet described in published literature, software must be made available to editors and reviewers. We strongly encourage code deposition in a community repository (e.g. GitHub). See the Nature Portfolio guidelines for submitting code & software for further information.

Data

Policy information about availability of data

All manuscripts must include a data availability statement. This statement should provide the following information, where applicable:

- Accession codes, unique identifiers, or web links for publicly available datasets
- A description of any restrictions on data availability
- For clinical datasets or third party data, please ensure that the statement adheres to our policy

Proteomics data have been deposited on PRIDE. The source data used to generate plots are provided as a Source Data file.

Human research participants

Policy information about studies involving human research participants and Sex and Gender in Research.

Reporting on sex and gender	No human research participants are involved in this work.
Population characteristics	No human research participants are involved in this work.
Recruitment	No human research participants are involved in this work.
Ethics oversight	No human research participants are involved in this work.

Note that full information on the approval of the study protocol must also be provided in the manuscript.

Field-specific reporting

Please select the one below that is the best fit for your research. If you are not sure, read the appropriate sections before making your selection.

☒ Life sciences ☐ Behavioural & social sciences ☐ Ecological, evolutionary & environmental sciences

For a reference copy of the document with all sections, see nature.com/documents/nr-reporting-summary-flat.pdf

Life sciences study design

All studies must disclose on these points even when the disclosure is negative.

Sample size	Biological and technical replicates were performed for each experiment. Experiments were repeated to enable calculation of standard deviations and for statistical analyses.
Data exclusions	No data were excluded from the analysis.
Replication	All attempts at replication were successful.
Randomization	Randomization was not relevant, and not performed in this work.
Blinding	Blinding was not relevant, and not performed in this work.

Reporting for specific materials, systems and methods

We require information from authors about some types of materials, experimental systems and methods used in many studies. Here, indicate whether each material, system or method listed is relevant to your study. If you are not sure if a list item applies to your research, read the appropriate section before selecting a response.

Materials & experimental systems

n/a	Involved in the study
☒	☐ Antibodies
☒	☐ Eukaryotic cell lines
☒	☐ Palaeontology and archaeology
☒	☐ Animals and other organisms
☒	☐ Clinical data
☒	☐ Dual use research of concern

Methods

n/a	Involved in the study
☒	☐ ChIP-seq
☐	☒ Flow cytometry
☒	☐ MRI-based neuroimaging

Flow Cytometry

Plots

Confirm that:

☒ The axis labels state the marker and fluorochrome used (e.g. CD4-FITC).

☒ The axis scales are clearly visible. Include numbers along axes only for bottom left plot of group (a 'group' is an analysis of identical markers).

☒ All plots are contour plots with outliers or pseudocolor plots.

☒ A numerical value for number of cells or percentage (with statistics) is provided.

Methodology

Sample preparation	Log phase cultures of Enterococcus faecalis strain OG1RF were diluted to OD600 of 0.1 in 10% TSB in the presence of absence of menadione and incubated for 30 min at 37 °C followed by the addition of CellROX green (final 0.5 µM) for a further 30mins at 37 °C in the dark with shaking at 180 rpm.
Instrument	Attune Nxt with a HTS autosampler system (Thermo Fisher Scientific)
Software	Attune Nxt v4.2.0 (Thermo Fisher Scientific)
Cell population abundance	No sorting was performed. Fluorescence was determined for the entire gated population.
Gating strategy	Gating was set using unstained samples for the bacterial population by forward-scatter (FSC; correlates with cell size) and side-scatter (SSC; correlates with cell internal granularity) of light and to determine background fluorescence.

☒ Tick this box to confirm that a figure exemplifying the gating strategy is provided in the Supplementary Information.
